# Exaptation of Bornavirus-Like Nucleoprotein Elements in Afrotherians

**DOI:** 10.1371/journal.ppat.1005785

**Published:** 2016-08-12

**Authors:** Yuki Kobayashi, Masayuki Horie, Ayumi Nakano, Koichi Murata, Takuya Itou, Yoshiyuki Suzuki

**Affiliations:** 1 Nihon University Veterinary Research Center, Fujisawa, Kanagawa, Japan; 2 Transboundary Animal Diseases Research Center, Joint Faculty of Veterinary Medicine, Kagoshima University, Kagoshima, Japan; 3 United Graduate School of Veterinary Science, Yamaguchi University, Yamaguchi, Japan; 4 Department of Animal Resource Science, College of Bioresource Sciences, Nihon Universitym, Fujisawa, Kanagawa, Japan; 5 Graduate School of Natural Sciences, Nagoya City University, Nagoya, Aichi, Japan; University of Utah, UNITED STATES

## Abstract

Endogenous bornavirus-like nucleoprotein elements (EBLNs), the nucleotide sequence elements derived from the nucleoprotein gene of ancient bornavirus-like viruses, have been identified in many animal genomes. Here we show evidence that EBLNs encode functional proteins in their host. Some afrotherian EBLNs were observed to have been maintained for more than 83.3 million years under negative selection. Splice variants were expressed from the genomic loci of EBLNs in elephant, and some were translated into proteins. The EBLN proteins appeared to be localized to the rough endoplasmic reticulum in African elephant cells, in contrast to the nuclear localization of bornavirus N. These observations suggest that afrotherian EBLNs have acquired a novel function in their host. Interestingly, genomic sequences of the first exon and its flanking regions in these EBLN loci were homologous to those of transmembrane protein 106B (TMEM106B). The upstream region of the first exon in the EBLN loci exhibited a promoter activity, suggesting that the ability of these EBLNs to be transcribed in the host cell was gained through capturing a partial duplicate of TMEM106B. In conclusion, our results strongly support for exaptation of EBLNs to encode host proteins in afrotherians.

## Author Summary

Endogenous retroviruses are representative of endogenous viral elements (EVEs), which are known to have occasionally served as the source of evolutionary innovations of the host. Endogenous bornavirus-like nucleoprotein element (EBLN) was the first EVE identified in mammalian genomes to have been derived from a non-retroviral RNA virus. Here we show evidence that EBLNs that were integrated into afrotherian genomes more than 83.3 million years ago have gained novel protein functions associated with rough endoplasmic reticulum in afrotherians. In the amino acid sequence of EBLN proteins, negative selection appeared to have operated more strongly on hydrophilic regions than on hydrophobic regions, suggesting that EBLN proteins may interact with other molecules in their host cells. In addition, we clarified the mechanism how EBLNs have acquired an ability to be transcribed in the host cell; they captured a partial duplicate of an intrinsic gene, transmembrane protein 106B, which retained an intrinsic promoter activity. Our findings suggest that not only retroviral EVEs but also non-retroviral EVEs may have contributed to the host evolution.

## Introduction

Many eukaryotic genomes contain endogenous viral elements (EVEs), which are derived from viruses [1,2]. Retroviruses are known as a major source of EVEs, such that approximately 8% of the human genome consists of endogenous retroviruses (ERVs) [3]. These EVEs not only serve as molecular fossil records representing the development of ancient to modern relationships between retroviruses and hosts, but also occasionally contribute to the evolution of hosts through exaptation [4–7]. For example, the *Syncytin* genes derived from envelope genes of retroviruses are involved in placentation in mammals [6]. In addition, some ERVs are involved in EVE-derived immunity (EDI), which acts as an anti-virus factor against exogenous retrovirus infections [4,5,7].

Recently, EVEs derived from non-retroviral DNA and RNA viruses were discovered in many eukaryotic genomes [8–12]. An endogenous bornavirus-like element (EBL) was the first EVE identified to be derived from a non-retroviral RNA virus in mammalian genomes [10]. EBLs are most closely related to bornavirus, which is a mononegavirus encoding a nucleoprotein (N), phosphoprotein (P), matrix protein (M), glycoprotein (G), RNA-dependent RNA polymerase (L), and accessory protein (X) [13,14]. To date EBLs derived from N, M, G, and L genes have been identified and designated as EBLN, EBLM, EBLG, and EBLL, respectively [9]. Some EBL sequences are apparently initiated with a bornavirus transcription start site and ended with a polyA and flanked by target-site duplications (TSDs), suggesting that they were generated through the LINE-1 machinery [8–12].

EBLNs are found in the genomes of diverse animals including snakes, turtles, moles, rodents, primates, and afrotherians [8–17]. ORFs found in EBLNs are in most cases fragmented by premature termination codons, although occasionally EBLNs with relatively long ORFs have been identified. Haplorhini primates have maintained an EBLN encoding 366 amino acids (aa), which is similar in length to bornavirus N (370 aa), for more than 40 million years (MY). However, no natural selection has been detected on primate EBLNs at the amino acid sequence level [15]. Thirteen-lined ground squirrel (*Ictidomys tridecemlineatus*) has harbored an EBLN (itEBLN) encoding 207 aa for ~0.3 MY [18]. A recombinant itEBLN protein conferred resistance to bornavirus infection on human cells by being incorporated into bornavirus particles as incompetent nucleoproteins [19], suggesting a possibility that itEBLN might act as an EDI protein, which should be tested in thirteen-lined ground squirrel. Overall, there is little evidence indicating that EBLNs encode a functional protein in their hosts.

Here, in a search for EBLNs that have been co-opted to encode functional proteins in the host, we investigated afrotherian EBLNs containing relatively long ORFs. Afrotherians are a diverse group of mammals that originated in Africa 83.3 MY ago (MYA) [20]. The superorder Afrotheria includes orders Proboscidea (e.g., elephant), Sirenia (e.g., dugong and manatee), Hyracoidea (e.g., hyracoid), Macroscelidea (e.g., elephant-shrew), Tubulidentata (e.g., aardvark), and Tenrecoidea (e.g., tenrec and golden mole) [21]. We present evidence for the existence of EBLNs encoding functional proteins and their evolutionary mechanisms in afrotherians.

## Results

### Finding afrotherian EBLNs encoding functional proteins

Using the amino acid sequence of bornavirus N (strain name: H1499, accession number: AY374520) as the query, a tBLASTn search was conducted against whole genome shotgun sequences of afrotherians (taxid: 311790) on June 24^th^, 2015. A total of 25 EBLNs were identified from African elephant (*Loxodonta africana*), rock hyrax (*Procavia capensi*), Florida manatee (*Trichechus manatus latirostris*), Cape golden mole (*Chrysochloris asiatica*), Cape elephant shrew (*Elephantulus edwardii*), and aardvark (*Orycteropus afer*), with an e-value threshold of E-20 (S1 Table). Two EBLNs each in African elephant, rock hyrax, Florida manatee, Cape elephant shrew, and aardvark contained relatively long ORFs encoding 341–350 aa, which were similar in length to bornavirus N (370 aa) (S1 Fig). Although two EBLNs were identified in Cape golden mole, the ORF of one EBLN (Ca/AMDV01031468) was separated into two fragments encoding 196 aa and 142 aa. The other EBLN (Ca/AMDV01100225) contained an ORF encoding 314 aa, which showed the highest amino acid sequence identity of 53% to bornavirus N among the afrotherian EBLNs identified in this study.

**Fig 1:**
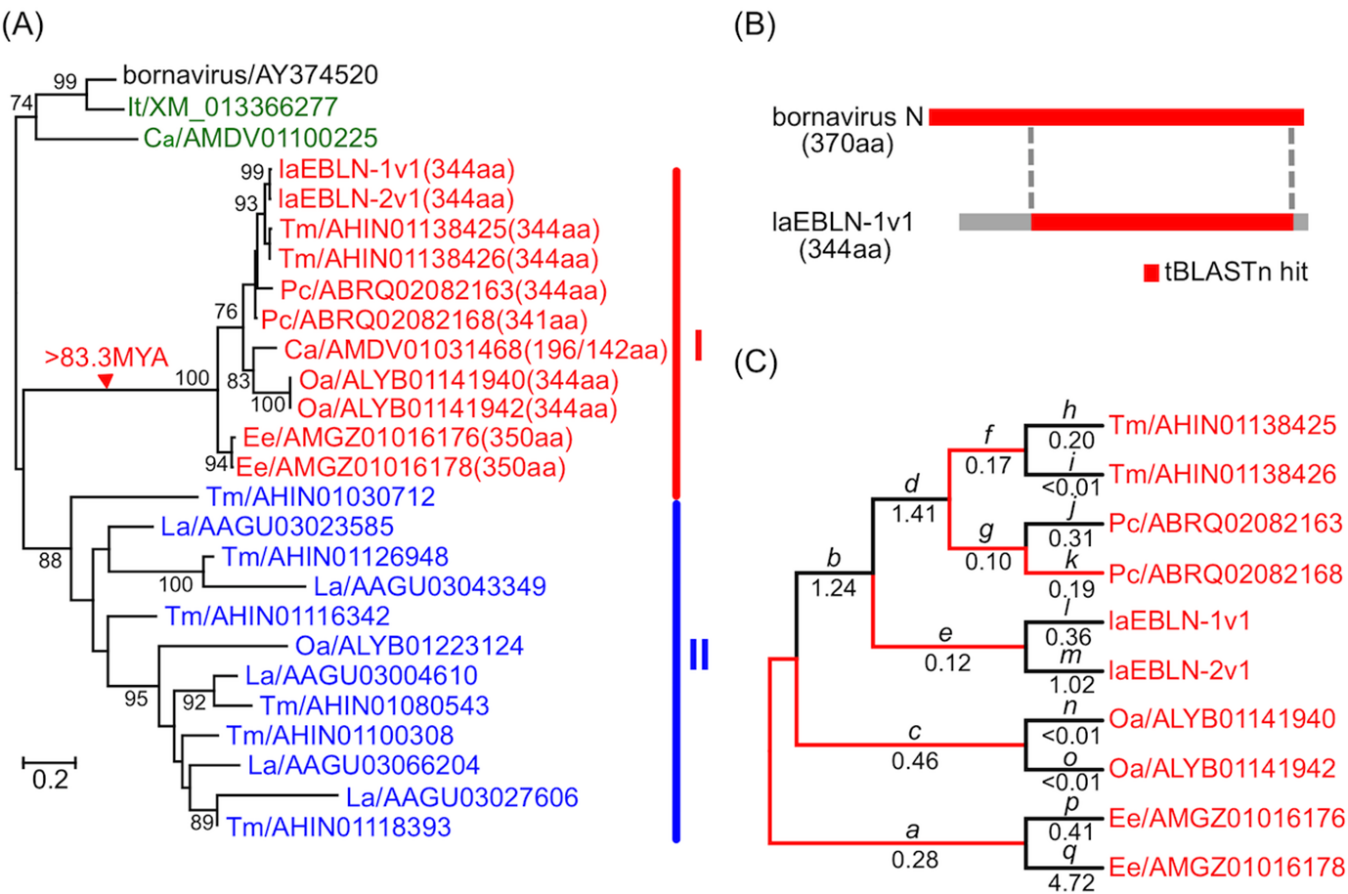
Molecular evolutionary analyses of afrotherian EBLNs. (A) Phylogenetic tree for EBLNs and bornavirus N constructed by the neighnour-joing method based on the amino acid sequences. Each sequence is described as abbreviation of the name of host species or virus/accession number (number of amino acid residues for cluster I EBLNs), except for cluster I EBLNs identified in the genome of *Loxodonta africana*, that is laEBLN-1v1 and laEBLN-2v1. La: *Loxodonta Africana*, It: *Ictidomys tridecemlineatus*, Tm: *Trichechus manatus latirostris*, Pc: *Procavia capensis*, Ca: *Chrysochloris asiatica*, Oa: *Orycteropus afer*, and Ee: *Elephantulus edwardii*. Red-, blue-, and green-letters are used for describing the EBLNs belonging to cluster I, cluster II, and others, respectively. Bootstrap values > 70% are shown for interior branches. A red-triangle points to the interior branch, on which cluster I EBLN was established in afrotherian genomes. (B) Comparison of ORFs (red) contained in bornavirus N and laEBLN-1v1. Boundaries of the homologous regions between bornavirus N and laEBLN-1v1 identified by tBLASTn search with E-20 are connected with dotted-lines. (C) The *d*_N_/*d*_S_ analysis for the ORFs encoded by cluster I EBLNs. The average value of *d*_N_/*d*_S_ was 0.34 over the entire phylogenetic tree, where negative seletion was detected with *p* = 2.1 × 10^−24^ by the likelihood ratio test. Estimate of *d*_N_/*d*_S_ value is shown below each branch, which is colored red when negative selection was detected with *p* < 0.05 by the likelihood ratio test. Detailed information on the estimates for branches *a-q* is shown in S2 Table.

In the phylogenetic tree of afrotherian EBLNs and bornavirus N (Fig 1A and S2 Fig), all EBLNs encoding 341–350 aa as well as the EBLN of Cape golden mole with separated ORFs (Ca/AMDV01031468) formed a monophyletic cluster (cluster I). The topology of cluster I EBLNs in Fig 1A reflected that of the host species [21]. In addition, the flanking genomic nucleotide sequences of cluster I EBLNs were alignable between afrotherians (S3 Fig), suggesting that cluster I EBLNs originated from a single integration event of viral N gene into the genome of ancestral afrotherians more than 83.3 MYA (Fig 1A) [20]. Afrotherian EBLNs with fragmented ORFs formed another cluster (cluster II), which contained EBLNs from African elephant, Folorida manatee, and aardvark. When EBLN sequences identified from non-afrotherian species were added to the phylogenetic tree, cluster I EBLNs and cluster II EBLNs were still monophyletic and constituted a larger cluster with Strepsirrhini EBLNs (S4 Fig). These results suggest that afrotherians have suffered from infection by bornavirus-like virus from the time of their origin and that colonizaiton of EBLNs has occurred at least twice during evolution of afrotherians.

When the degree of sequence divergence (branch lengths) was compared between cluster I and cluster II EBLNs, it was evident that the amino acid sequences encoded by the former EBLNs evolved more slowly than those encoded by the latter (Fig 1A and S2 Fig), suggesting the functionality of amino acid sequences encoded by cluster I EBLNs. Indeed, the average *d*_N_/*d*_S_ ratio was estimated to be 0.34 and negative selection was detected (*p* = 2.1 × 10^−24^ by the likelihood ratio test) for the entire phylogenetic tree of cluster I EBLNs (Fig 1C). In addition, when the *d*_N_/*d*_S_ ratio was estimated at each branch of the phylogenetic tree for cluster I EBLNs, the *d*_N_/*d*_S_ ratio was smaller than one and negative selection was detected (*p* < 0.05 by the likelihood ratio test) at most of the branches (Fig 1C and S2 Table). These results indicate that cluster I EBLNs may encode functional proteins in afrotherians.

### mRNA expression from EBLN loci in elephant

**Fig 2:**
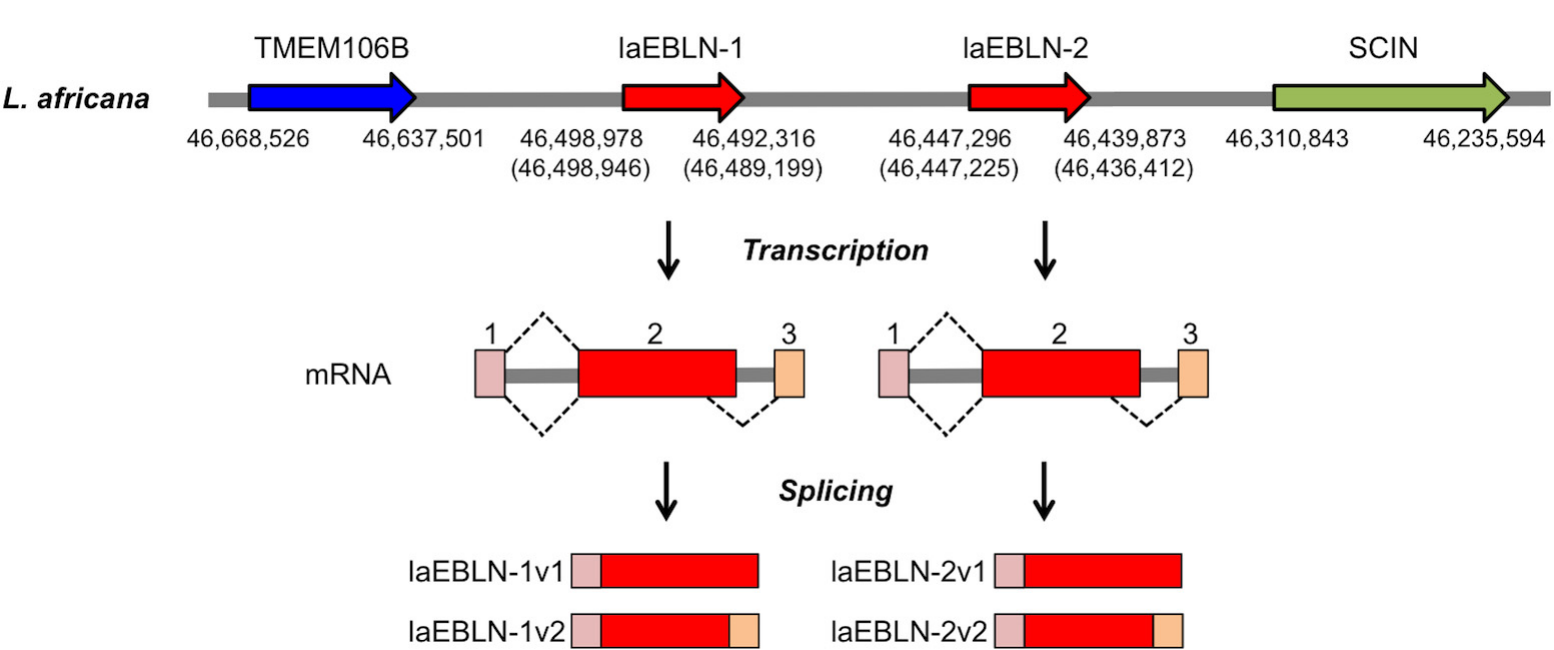
Gene structures at laEBLN loci in the genome of *L*. *africana*. Genomic locations of laEBLN-1 and laEBLN-2 (red arrows) and their flanking genes, TMEM106B (blue arrow) and SCIN (green arrow), in the genome of *L*. *africana* (Loxafr 3.0, scaffold 5). Numbers under the arrows indicate the start and end positions of mRNAs for SCIN, laEBLN-1v1(1v2), laEBLN-2v1(2v2), and TMEM106B. Exons and introns at the laEBLN loci are represented by filled boxes and gray bars, respectively.

In cluster I, there were two EBLNs obtained from African elephant, named laEBLN-1 (contig accession number: AAGU03015684) and laEBLN-2 (contig accession number: AAGU03015682) (Fig 1A). These EBLNs contained ORFs encoding 344 aa of identical sequences except for a single site (S1 Fig), each of which was 33% identical with the sequence of bornavirus N (S5 Fig). laEBLN-1 and laEBLN-2 were mapped ~45 kb apart on the same chromosome of African elephant (Loxafr3.0, supercontig 5) (Fig 2 and S3 Table). The genomic region encompassing these loci were flanked by transmembrane protein 106B (TMEM106B) (accession number: XM_003407108) and scinderin (SCIN) (accession number: XM_003407107). The syntenic relationship among TMEM106B, two copies of cluster I EBLNs, and SCIN as above was also observed in the genome of Florida manatee (S6 Fig). Although the syntenic relationship of TMEM106B and SCIN was observed in the genomes of many vertebrates (S4 Table), no sequence element related to laEBLN-1 or laEBLN-2 was identified between them in non-afrotherian genomes (S7 Fig), supporting the notion that the integration event of cluster I EBLNs took place on the lineage of afrotherians.

**Fig 3:**
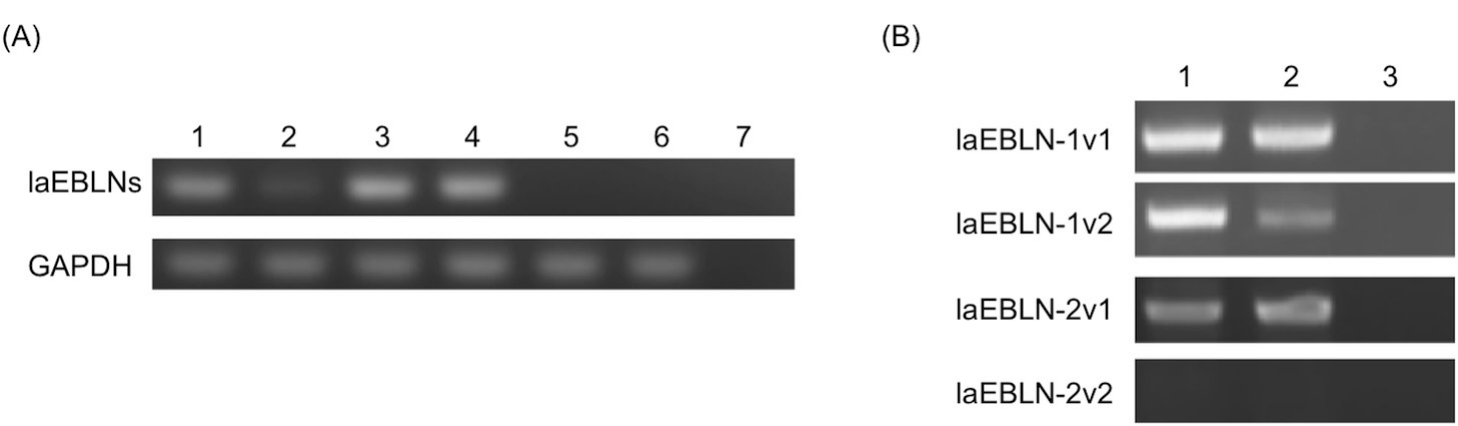
mRNA expression of laEBLNs in elephant tissues and cell lines. (A) RT-PCR for laEBLN-1 and laEBLN-2 with the primer pair a/b and for GAPDH (See S8 Fig and S5 Table for details of the primer pairs). Total RNAs were extracted from the liver (lane 1) and the muscle (lane 2) of *E*. *maximus*, LACF-NANAI (lane 3), LACF-NANAII (lane 4), BHK cells (lane 5), 293F cells (lane 6), and distilled water as negative control (lane 7). (B) RT-PCR with the primer pairs c/e for laEBLN-1v1, d/e for laEBLN-1v2, c/f for laEBLN-2v1, and d/f for laEBLN-2v2 (See S8 Fig and S5 Table for details of the primer pairs). Total RNAs were extracted from LACF-NANAI (lane 1), LACF-NANAII (lane 2), and distilled water as negative control (lane 3).

To examine whether laEBLN-1 and laEBLN-2 were transcribed into mRNAs, RT-PCR was conducted with a set of primers that was designed to amplify DNA fragments of the same size from these EBLNs (S8 Fig and S5 Table). A DNA fragment of the expected size was amplified from total RNA extracted from liver and muscle tissues from an adult male Asian elephant (*Elephas maximus*) and cell lines established from African elephant ear (LACF-NANAI) and gum (LACF-NANAII) tissues (Fig 3A and S9 Fig). These results suggest that mRNAs containing laEBLN-1 and/or laEBLN-2 and their orthologues are transcribed ubiquitously in African and Asian elephants, respectively. Complete nucleotide sequences of mRNAs containing laEBLN-1 and laEBLN-2, named laEBLN-1v1 (accrssion number: LC093509) and laEBLN-2v1 (accession number: LC093510), respectively, were determined by 5’ and 3’ RACEs. Both mRNAs consisted of two exons, 1 and 2, where EBLN was embedded in exon 2. Exon 1 and its upstream region, exon 2 and its downstream region, and the intron were all homologous between the genomic loci for laEBLN-1v1 and laEBLN-2v1 (S10 Fig), suggesting that these loci were generated through gene duplication at the DNA level.

It should be noted that mRNAs apparently transcribed from the same genomic loci as laEBLN-1v1 and laEBLN-2v1 but processed in alternative splicing forms have been deposited in the RNAseq database for African elephant (BioSample: SAMN02953622), and were named laEBLN-1v2 (accession number: LOC104845604) and laEBLN-2v2 (accession number: LOC104845603), respectively, in this study (Fig 2). Both of the alternatively spliced forms consisted of three exons, 1, 2, and 3. Exon 1 and a part of exon 2 were shared between laEBLN-1v1 and laEBLN-1v2 and between laEBLN-2v1 and laEBLN-2v2 (S11 Fig). When the mRNA expression of laEBLN-1v1, laEBLN-1v2, laEBLN-2v1, and laEBLN-2v2 was examined separately by RT-PCR with four sets of primers that were designed to amplify individual mRNAs (S8 Fig and S5 Table), laEBLN-1v1, laEBLN-1v2, and laEBLN-2v1 were detected in both LACF-NANAI and LACF-NANAII (Fig 3B). In contrast, laEBLN-2v2 was not detected in either cell line, suggesting a differentiation in expression patterns between splice variants.

### Protein expression of laEBLNs in elephant cells

**Fig 4:**
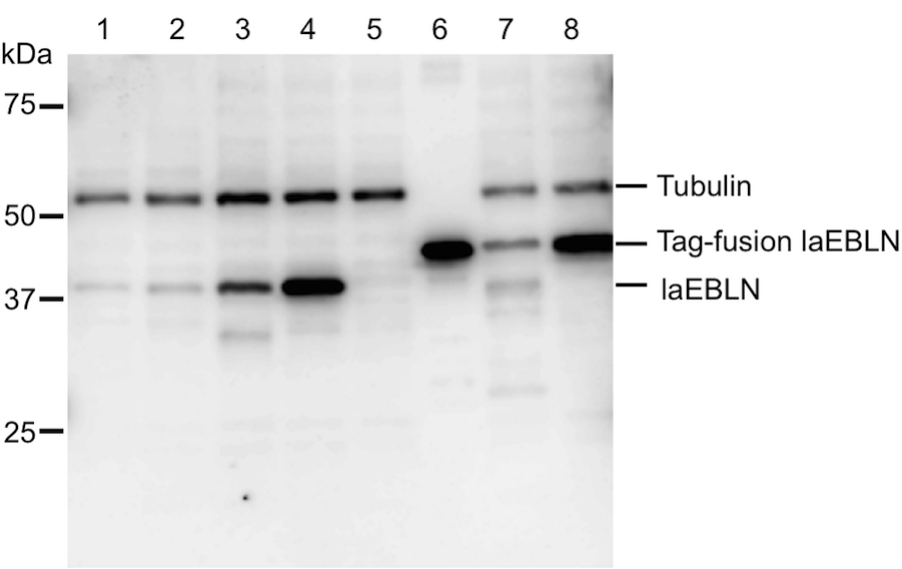
Protein expression from laEBLN mRNAs in elephant cells. Lane 1: LACF-NANAI, lane 2: LACF-NANAII, lane 3: 293F cells with laEBLN-1v1 expression plasmid, lane 4: 293F cells with laEBLN-1v2 expression plasmid, lane 5: 293F cells, lane 6: rlaEBLN-1v1 expressed in *E*. *coli* (15 ng/lane), lane 7: 293F cells with FLAG-tagged laEBLN-1v1 expression plasmid, and lane 8: 293F cells with His-tagged laEBLN-1v2 expression plasmid.

The expected molecular weights of the proteins encoded by laEBLN-1v1, laEBLN-1v2, laEBLN-2v1, and laEBLN-2v2 were 38,888 Da, 39,670 Da, 38,860 Da, and 39,642 Da, respectively. To examine whether proteins were expressed from these mRNAs, rabbits were immunized with a recombinant laEBLN-1v1 (rlaEBLN-1v1) protein expressed in *E*. *coli* to induce polyclonal antibodies, which were confirmed to react with rlaEBLN-1v1 (Fig 4, lane 6). The rabbit polyclonal antiserum was also found to react with both of laEBLN-1v1 and laEBLN-1v2 proteins expressed in 293F cells in the western blot analysis (Fig 4, lanes 3 and 4). When the western blot analysis using the rabbit polyclonal antiserum was performed on the whole protein extracts from LACF-NANAI and LACF-NANAII, a single band was observed at ~38 kDa (Fig 4, lanes 1 and 2), indicating protein expression from some laEBLN mRNAs in African elephant cells.

**Fig 5:**
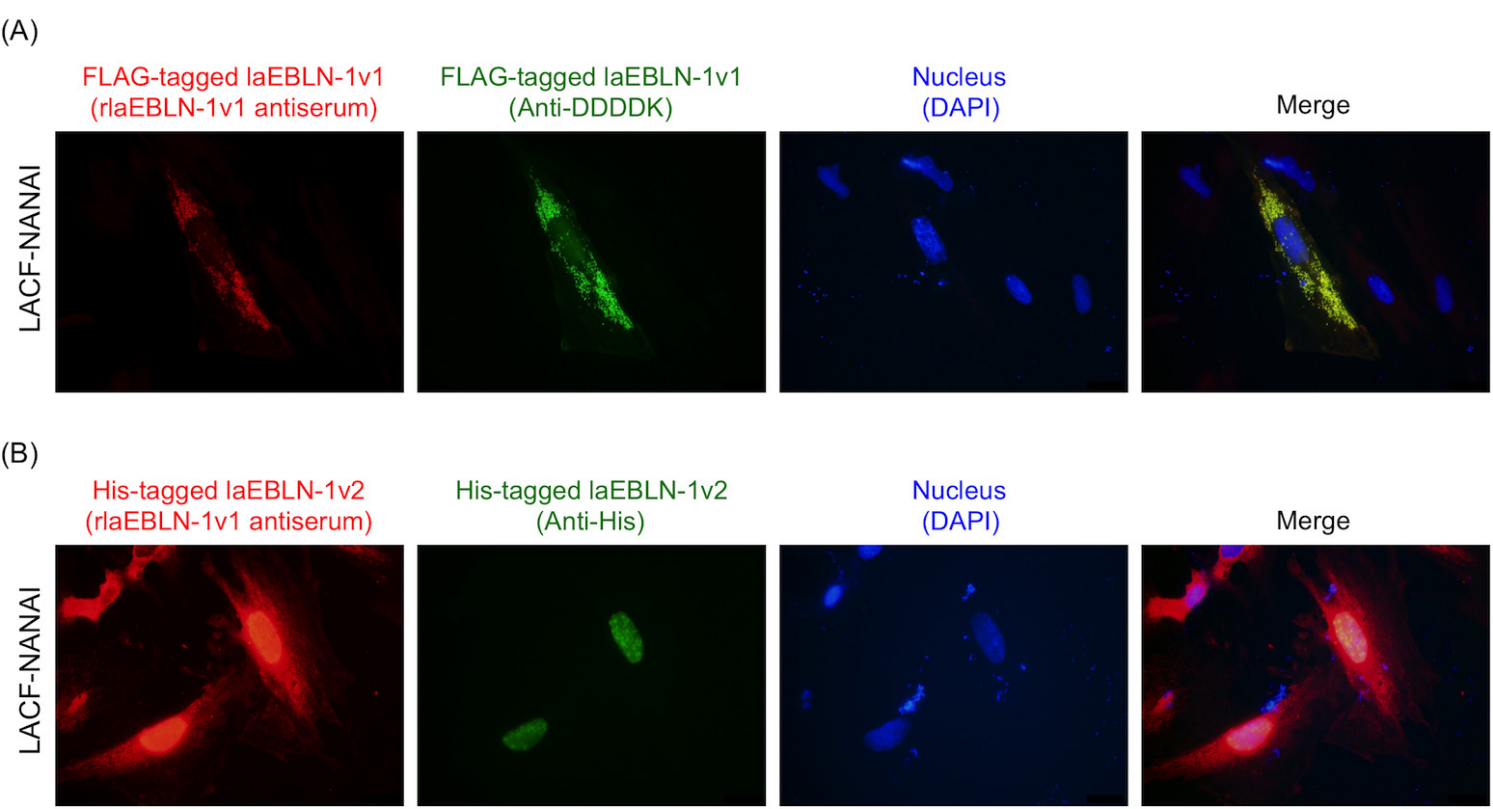
Cellular localization of laEBLN-1v1 and laEBLN-1v2 proteins expressed in LACF-NANAI. Immunohistochemical staining of (A) FLAG-tagged laEBLN-1v1 and (B) His-tagged laEBLN-1v2 proteins. First-antibodies or DAPI used for immunohistochemical staining are shown in parentheses.

To examine the cellular localization of each variant protein, FLAG-tagged laEBLN-1v1 and His-tagged laEBLN-1v2 proteins, which were confirmed to react with rabbit polyclonal antibodies (Fig 4, lanes 7 and 8), were expressed in LACF-NANAI, and were stained with anti-DDDDK- and anti-His-mouse monoclonal antibodies. Note that in the bioinformatic analysis of the amino acid sequences of laEBLN-1v1 and laEBLN-1v2 proteins using PSORT II [22], the former was predicted to be localized to the cytoplasm, whereas the latter to the nucleus (S6 Table). Consistently, the signal of FLAG-tagged laEBLN-1v1 protein was detected in the cytoplasm (Fig 5A), whereas the singal of His-tagged laEBLN-1v2 protein in the nucleus (Fig 5B). Similar cellular localizations of laEBLN proteins were also observed in 293F cells (S12 Fig), suggesting that laEBLN-1v1 and laEBLN-1v2 proteins may play different functions. It should be noted that bornavirus N is known to be localized to the nucleus of infected cells. It is therefore conceivable that the protein product of laEBLN-1v1 may have acquired a novel function in afrotherians.

**Fig 6:**
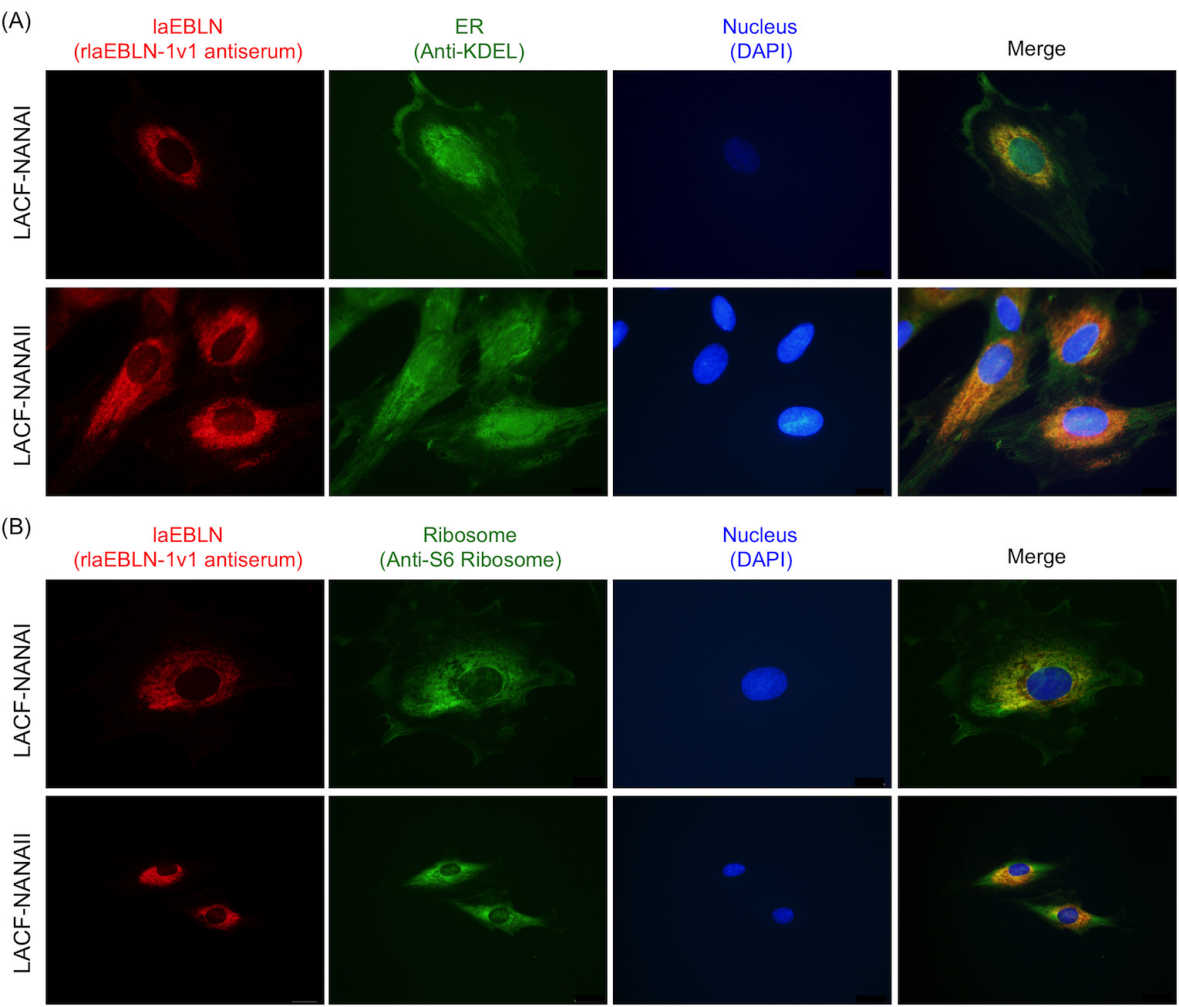
Cellular localization of endogenous laEBLN proteins in elephant cells. Immunohistochemical staining with rabbit polyclonal antiserum against rlaEBLN-1v1 protein (red) and with (A) anti-KDEL for ER (green) or (B) anti-S6 ribosomal protein antibodies (green).

We then stained LACF-NANAI and LACF-NANAII with rabbit polyclonal antibodies to identify the cellular localizations of endogenous protein products from laEBLN mRNAs. Positive signals were detected around the nucleus in the cytoplasm, co-localizing with the perinuclear part of the endoplasmic reticulum (ER) (Fig 6A) and, in particular, with the ribosome (Fig 6B), suggesting that laEBLN proteins were associated with the rough ER (rER) in African elephant cells. The presence and absence of positive signals in the cytoplasm and nucleus, respectively, may reflect relative abundance of laEBLN proteins in African elephant cells. Assuming that the rabbit polyclonal antibodies could recognize all of the protein products from laEBLN-1v1, laEBLN-1v2, laEBLN-2v1, and laEBLN-2v2 and the cellular localizations of the protein products from laEBLN-1v1 and laEBLN-2v1 were cytoplasmic and those from laEBLN-1v2 and laEBLN-2v2 were nuclear, the observed pattern of positive signals may be consistent with the result obtained above that mRNA expression of laEBLN-2v2 was not detected in African elephant cells (Fig 3B).

### Evolutionary mechanism for protein functionalization in afrotherian EBLNs

**Fig 7:**
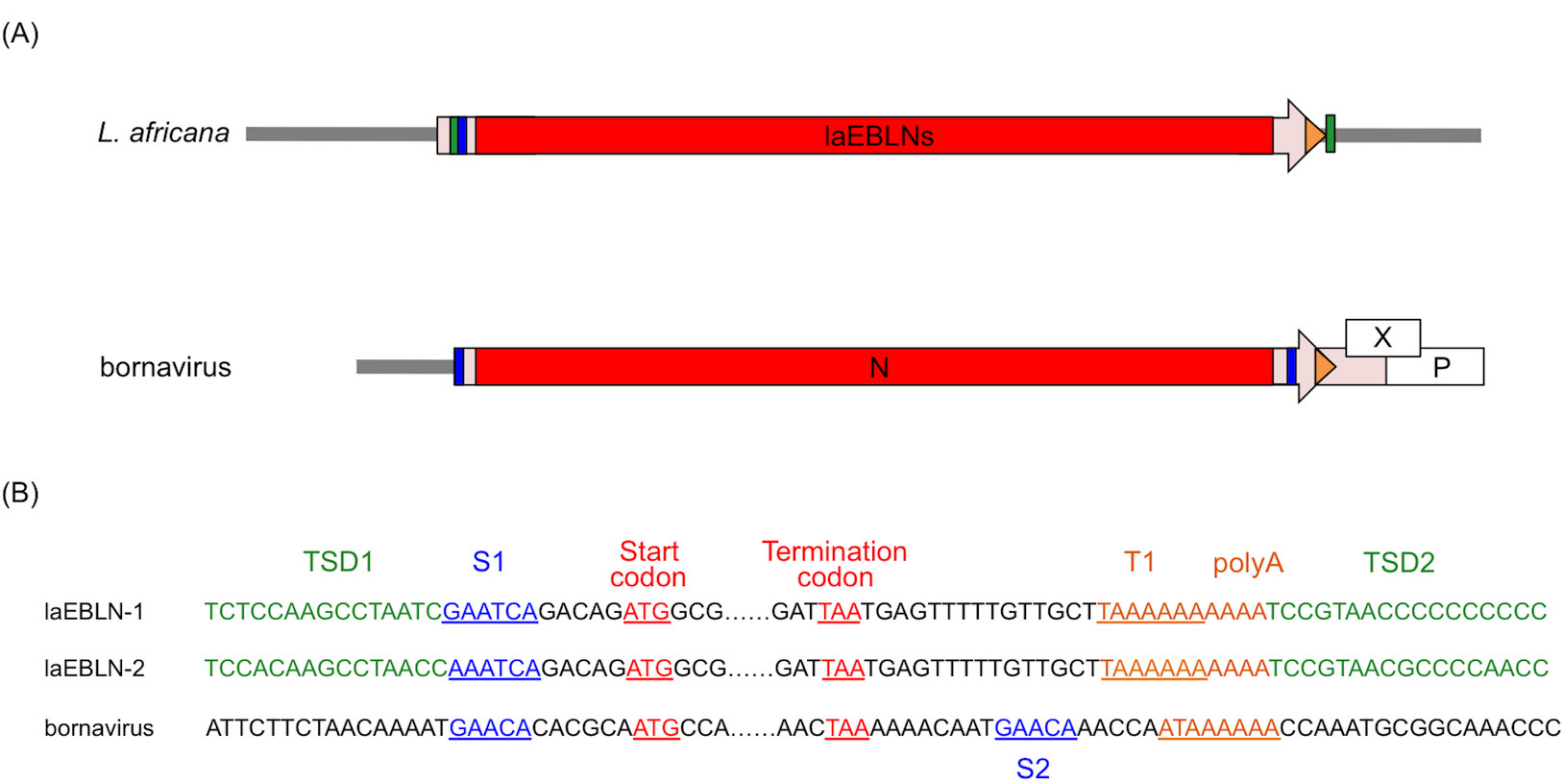
Nucleotide sequences of target site duplications (TSDs) found in laEBLN loci. (A) Schematic diagram of exon 2 (arrow) of laEBLN-1v1 and laEBLN-2v1 in *L*. *africana* genome, and N mRNA and its surrounding region in bornavirus genome. Coding and non-coding regions of mRNA are shown in red and pink, respectively. Positions of TSDs, bornavirus transcription start sites 1 (S1) and 2 (S2), transcription stop site (T1) and polyA are colored green, blue, and orange, respectively. (B) Nucleotide sequences from the start codon to the upstream TSD1 and from the termination codon to the downstream TSD2 in laEBLN-1 and laEBLN-2, together with the nucleotide sequences at the corresponding regions of bornavirus N.

In the genomic sequence of African elephant, laEBLN-1 and laEBLN-2 were both followed by transcription stop site (T1) and polyA (Fig 7). The sequence regions encompassing laEBLN-1 and its polyA and laEBLN-2 and its polyA were both flanked by TSDs with similar sequences. In addition, a 6 nt sequence related to a transcription start site (S1) of bornavirus was observed immediately downstream of the 5’ TSD (TSD1) and 5 nt upstream of the start codon in laEBLN-1. These observations indicate that laEBLN-1 and laEBLN-2 have originated from a common integration event of a reverse-transcribed mRNA for viral N gene through the LINE-1 machinery, similarly to the case for the EBLNs previously identified in other animal species [8,10].

**Fig 8:**
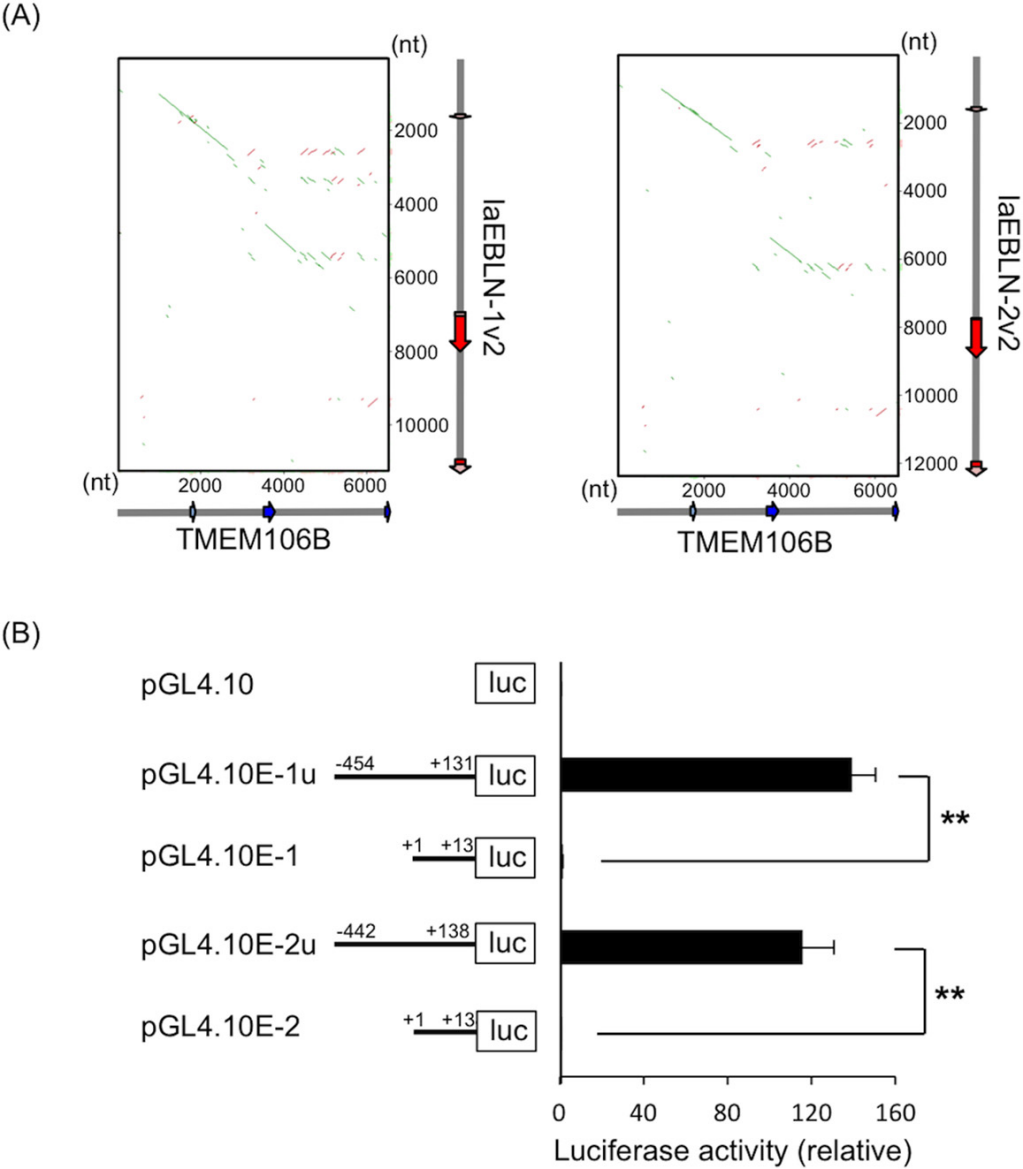
Promoter assay for exon 1 and its upstream region of laEBLN-1 and laEBLN-2. (A) Dot-plots between the genomic loci for TMEM106B and laEBLN-1v2 or laEBLN-2v2. The upstream 1,500 nt from exon 1 of these loci were also included in the analysis. (B) Relative luciferase activities. Numbers on black lines indicate the nucleotide positions counted from the transcription start site for laEBLN-1v1/1v2 and laEBLN-2v1/2v2. Error bars represent the standard error of the mean. **p < 0.01 by Student’s *t*-test.

Here it should be noted that bornavirus N mRNA does not contain an eukaryotic promoter sequence, and thus it is unclear how the integrated sequence element gained the ability to be transcribed in the host cell. Interestingly, exon 1 and its flanking regions in the genomic loci for laEBLN-1v1/1v2 and laEBLN-2v1/2v2 in African elephant were discovered to be homologous to those for TMEM106B (Fig 8A and S13 Fig). In particular, the 5’ splice site (GU) for the first intron of laEBLN-1v1/1v2 and laEBLN-2v1/2v2 appeared to be derived from the corresponding site of TMEM106B (S14 Fig). It was then hypothesized that laEBLN-1 and laEBLN-2 gained the ability to be transcribed in the host cell by capturing a partial duplicate of TMEM106B, which contained a copy of the promoter and transcription start site (TSS) for TMEM106B.

To test this hypothesis, we conducted a promoter assay by constructing a series of luciferase expression plasmids, in which the luciferase gene was placed downstream of (1) exon 1 of laEBLN-1v1/1v2 (pGL4.10E-1u), (2) exon 1 of laEBLN-2v1/2v2 (pGL4.10E-2), (3) exon 1 and upstream 454 nt of laEBLN-1v1/1v2 (pGL4.10E-1u), and (4) exon 1 and upstream 442 nt of laEBLN-2v1/2v2 (pGL4.10E-2u) (Fig 8B). Note that 454 nt and 442 nt upstream of exon 1 of laEBLN-1v1/1v2 and laEBLN-2v1/2v2, respectively, were homologous to the upstream sequence of exon 1 of TMEM106B (Fig 8A). It was observed that luciferase activities of pGL4.10E-1u and pGL4.10E-2u were ~100 times higher than those of pGL4.10E-1 and pGL4.10E-2 (*p* < 0.01 by Student’s *t* test), respectively (Fig 8B), supporting the above hypothesis.

## Discussion

In this study, we found that afrotherian EBLNs were clustered into two phylogenetically distinct classes, i.e., cluster I and cluster II EBLNs, with an exception of the EBLN of Cape golden mole encoding 314 aa. Cluster I EBLNs originated from a single integration event of N mRNA from a bornavirus-like virus into the ancestral genome of afrotherians through the LINE-1 machinery more than 83.3 MYA, which overlaps with the time period when LINE-1 was active in afrotherians [23–25]. On the other hand, cluster II EBLNs were observed only in the genomes of African elephant, Florida manatee, and aardvark, suggesting that the integration event of cluster II EBLNs into the afrotherian genomes may have taken place relatively recently compared to cluster I EBLNs.

Amino acid sequences encoded by relatively long ORFs in cluster I EBLNs have been negatively selected, suggesting that they were co-opted in afrotherians as functional proteins. In contrast, ORFs in cluster II EBLNs were fragmented and apparently have evolved without functional constraint at the amino acid sequence level. The difference in the fates of cluster I and cluster II EBLN ORFs may have stemmed from the presence or absence of a partial duplicate of TMEM106B upstream of EBLNs in the genome. The promoter and TSS of human TMEM106B are located in the CpG island (S15 Fig), which is known to be associated with ubiquitously expressed genes such as house keeping genes, and human TMEM106B mRNA has been reported to be expressed ubiquitously [26–29]. In the genome of African elephant, GC content in the 100 nt upstream of laEBLN-1v1/1v2 and laEBLN-2v1/2v2 is 65% and 61%, respectively, suggesting that the promoter and TSS of these mRNAs are also located in the CpG island. Indeed, the overall expression of laEBLN-1v1/1v2 and laEBLN-2v1/2v2 was ubiquitous in elephant. It is conceivable that the partial duplicate of TMEM106B provided cluster I EBLNs with an opportunity to be transcribed ubiquitously in afrotherians, which may have facilitated the EBLN proteins to acquire novel functions in the host before the occurrence of deleterious mutations in the ORF. It should be noted, however, that acquisition of intrinsic promoter and TSS may not be necessary for transcription of EBLNs in the host cell, because all of seven EBLNs in human were shown to be transcribed in some tissues although their association with intrinsic promoters and TSS has not been identified [10,30,31]. In addition, mRNAs containing the cluster II African elephant EBLN (La/AAGU03023585, RNAseq accession number: XM_010588552) and the Cape golden mole EBLN encoding 314 aa (Ca/AMDV01100225, RNAseq accession number: XM_006861214) were found in the RNAseq database. It is possible that mRNAs containing these EBLNs were not expressed in sufficiently large numbers of tissues for acquisition of novel functions in the host before the occurrence of deleterious mutations in the ORF. However, it should also be noted that the protein function once acquired by EBLNs can be lost during evolution of the hosts. In afrotherians, the ORF of cluster I EBLN in Cape golden mole (Ca/AMDV0103146) was separated into two fragments, and Lesser hedgehog tenrec (*Echionps telfairi*) contained only remnants of cluster I EBLNs (contig accession numbers: AAIY02040498 and AAIY02084943), which could not be detected by the tBLASTn search conducted in this study. Interestingly, these species are closely related as members of Tenrecoidea. These information may be useful for understanding the protein function of cluster I EBLNs in other species.

**Fig 9:**
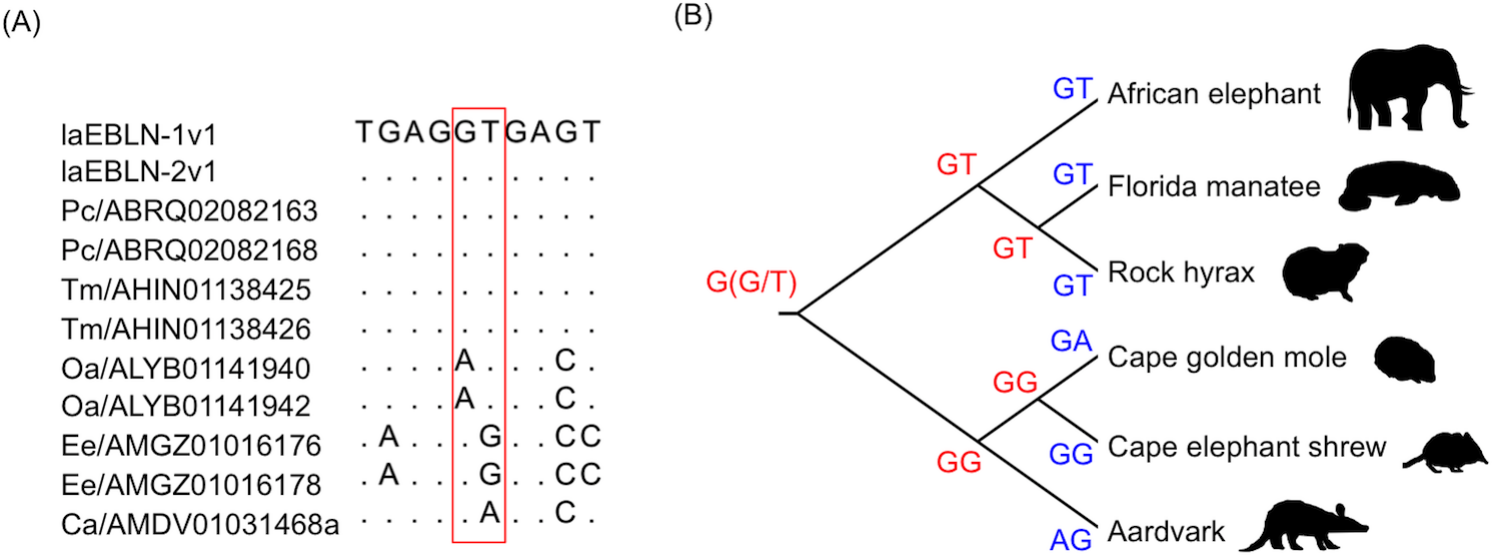
Splice donor sites in the second intron of afrotherian EBLNs. (A) Splice donor sites in the second intron of afrotherian EBLNs are surrounded by a red-boxed line. (B) Evolution of splice donor sites. Nucleotide sequences at the splice donor sites in current and ancestral afrotherian EBLNs are shown in blue and red, respectively.

Cluster I EBLNs were tandemly duplicated in afrotherian genomes, and were transcribed in alternatively spliced forms in African elephant, generating laEBLN-1v1, laEBLN-1v2, laEBLN-2v1, and laEBLN-2v2. Although these mRNAs as a whole appeared to be expressed ubiquitously, the expression profile of laEBLN-2v2 was diversified from those of laEBLN-1v1, laEBLN-1v2, and laEBLN-2v1. In addition, protein products of laEBLN-1v1 and laEBLN-2v1 were expected to be different from those of laEBLN-1v2 and laEBLN-2v2 at the C-terminal 20 aa, and the FLAG-tagged laEBLN-1v1 and His-tagged laEBLN-1v2 proteins showed different subcellular localizations in elephant cells. These observations were indicative of a functional differentiation among laEBLN proteins. Nevertheless, the splice donor site (GU) for the second intron to generate laEBLN-1v2 and laEBLN-2v2 were identified only in elephant, manatee, and hylax (Fig 9A), suggesting that expression of splice variants corresponding to laEBLN-1v2 and laEBLN-2v2 may be limited to these species. It was unclear whether these species gained or other species lost the splice variants because these scenarios were equally likely from the inference of ancestral sequences at the splice donor site according to the parsimony principle (Fig 9B).

It has been reported that the protein product of squirrel EBLN (itEBLN), which was identical to bornavirus N at 77% of amino acid sites, may suppress bornavirus infection by disrupting the function of N [19]. In contrast, bornavirus infection was not suppressed by the protein product of hsEBLN-1, which shared 41% of amino acid sequence with bornavirus N. The amino acid sequences encoded by afrotherian EBLNs were more divergent from bornavirus N than the sequence encoded by hsEBLN-1. In addition, endogenous laEBLN protein was observed to be localized to the rER in African elephant cells, which was in sharp contrast to the fact that bornavirus N is localized to the nucleus in infected cells [32,33]. These observations suggest that laEBLN proteins have acquired a novel function associated with rER in afrotherian cells.

**Fig 10:**
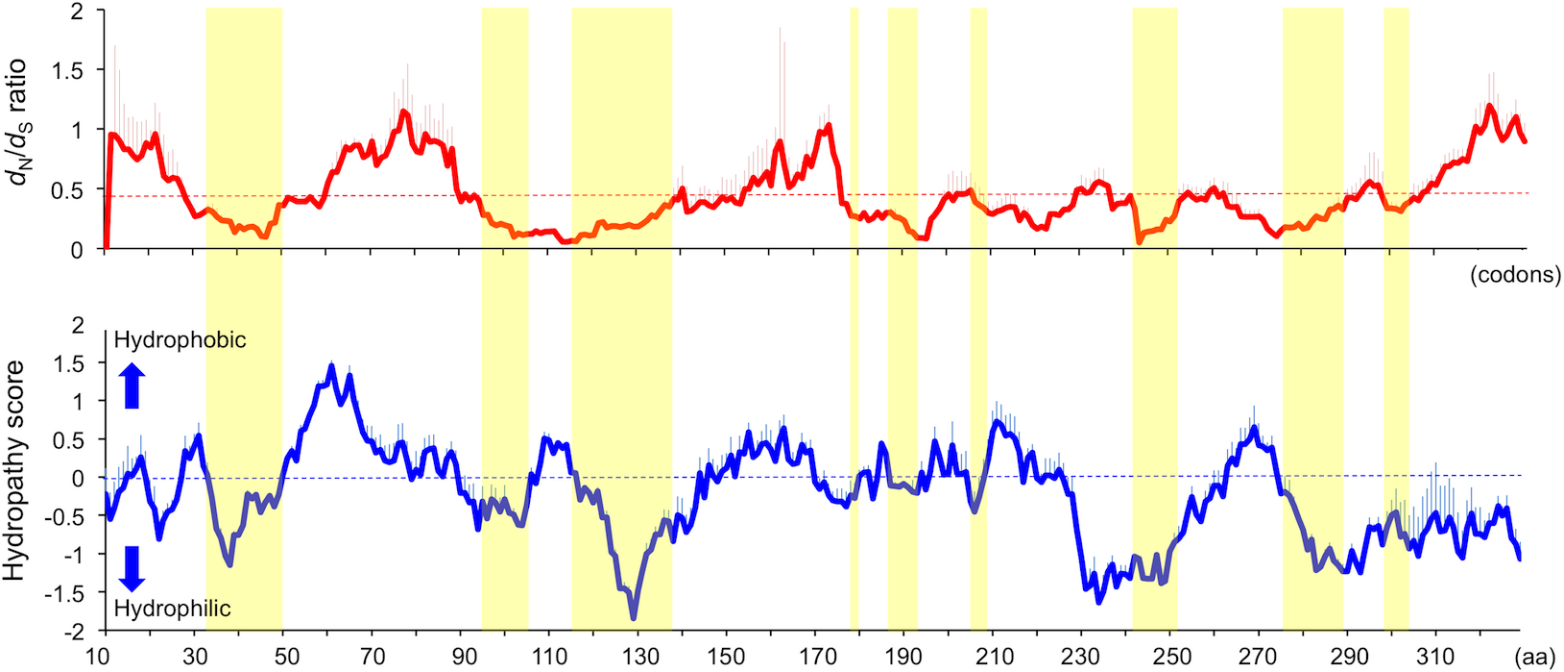
Window analyses of *d*_N_/*d*_S_ ratio and Kyte-Doolittle hydropathy plot along the ORFs encoded by afrotherian EBLNs. Solid red- and blue-lines show the average values of *d*_N_/*d*_S_ ratio and hydropathy score, respectively, at windows (= 20 codon sites) in ORFs encoded by cluster I EBLNs (variant 1). The red- and blue-longitudinal bars on the solid lines show the standard deviation of *d*_N_/*d*_S_ ratio and hydropathy score, respectively, at each window. Dotted red- and blue-lines show the average value of *d*_N_/*d*_S_ ratio (= 0.44) over all windows and the cut-off line of hydrophobic (hydropathy score > 0) and hydrophilic (hydropathy score < 0) regions, respectively. Yellow boxes indicate hydrophilic regions with *d*_N_/*d*_S_ ratio < 0.44.

The rER is associated with ribosomes and involved in the translation of cytoplasmic, secretory, and membrane proteins. There are mechanisms to deliver mRNAs from the cytoplasm to the rER membrane by the action of RNA-binding proteins, such as STAU1, STAU2, Pum1, and Pum2 [34–36]. Because mononegavirus N has an ability to bind to RNA and laEBLN proteins are localized to rER, it is interesting to assess the involvement of laEBLN proteins in mRNA delivery. The hydropathy plot of the amino acid sequence encoded by laEBLN-1v1 showed that laEBLN-1v1 protein may be soluble (Fig 10), which was consistent with the characteristic of the rlaEBLN-1v1 protein produced in *E*. *coli* (See Materials and Methods). In the window analysis of the *d*_N_/*d*_S_ ratio for the ORF in cluster I EBLNs, it appeared that negative selection has operated more strongly on hydrophilic regions (average *d*_N_/*d*_S_ ratio = 0.41) than on hydrophobic regions (average *d*_N_/*d*_S_ ratio = 0.48) (*p* < 0.05 by *z*-test where the variance of average *d*_N_/*d*_S_ ratio was estimated with bootstrap resampling of windows) (Fig 10), suggesting that the protein product of cluster I EBLNs may interact with other molecules and the interaction may be critical in afrotherians. It is interesting to clarify the function of laEBLN proteins to understand the impact of viruses on the evolution of their hosts.

## Materials and Methods

### Homology search

Afrotherian EBLNs were identified by a tBLASTn search using the amino acid sequence of bornavirus N (strain name: H1499; accession number: AY374520) as the query against the database of whole genome shotgun (WGS) sequences for afrotherians (taxid: 311790) on June 24^th^, 2015. Sequence hits with an e-value threshold of E-20 were identified as EBLNs. EBLNs in non-afrotherian species were also identified from the database of WGS sequences for vertebrates (taxid: 7742) on May 18^th^, 2016 by the same criterion as described above. Amino acid sequences encoded by EBLNs were subjected to HMMER for examining the existence of domains that have been deposited in the Pfam database.

### Sequence comparison

Multiple alignments of amino acid sequences for EBLNs and bornavirus N were made by mapping each of the amino acid sequences encoded by EBLNs onto that of bornavirus N (strain name: H1499; accession number: AY374520) according to the pairwise alignment of these sequences produced in the tBLASTn search (S1 File). In each EBLN sequence, the amino acid sites differentially aligned to bornavirus N in different tBLASTn hits were treated as gaps. In a dot-plot analysis, the genomic sequences of the genes of interest together with their upstream 1,500 nt were retrieved from Ensemble on 12^th^ May, 2015, and were compared using YASS [37]. Genomic sequences of the regions 46,200,000–46,700,000 in scaffold_5, supercontig loxFar3 of African elephant and 12,200,000–12,700,000 in chromosome 7, GRCh38 of human were subjected to synteny analysis using GeneMatcher (version 2.014) [38], in which BLASTn and tBLASTn searches were conducted for identifying homologous segments between these sequences.

### Molecular evolutionary analyses

The phylogenetic tree of EBLNs and bornavirus N was constructed by the maximum likelihood (ML) and nighbour-joing (NJ) methods with the partial deletion and pairwise deletion options in MEGA 6, respectively [39]. The JTT+G model was chosen as the best fit model of amino acid substitution with the smallest Bayesian information criterion score. The nearest-neighbor interchange was selected as the ML heustric method. The reliability of interior branches in the phylogenetic tree was assessed by computing the bootstrap probability with 1,000 resamplings. Divergence times between afrotherians were obtained from TimeTree [20].

The *d*_N_/*d*_S_ ratio for the entire phylogenetic tree as well as for each branch of cluster I EBLNs was estimated by the ML method using the codon substitution model in PAML ver. 4.0 [40]. The equilibrium codon frequencies were treated as free parameters. The selective neutrality was tested for the entire phylogenetic tree or for each branch by the likelihood ratio test. Window analysis of the *d*_N_/*d*_S_ ratio was conducted between laEBLN-1v1 and each of other cluster I EBLNs, i.e., Tm/AHIN01138425, Tm/AHIN01138426, Pc/ABRQ02082163, Pc/ABRQ02082168, Oa/ALYB01141940, Oa/ALYB01141942, Ee/AMGZ01016176, and Ee/AMGZ01016178, with a window size of 20 codons and a step size of 1 codon using ADAPTSITE [41].

Hydropathy scores along the amino acid sequences encoded by laEBLN-1v1, Tm/AHIN01138425, Tm/AHIN01138426, Pc/ABRQ02082163, Pc/ABRQ02082168, Oa/ALYB01141940, Oa/ALYB01141942, Ee/AMGZ01016176, and Ee/AMGZ01016178 were calculated as the Kyte and Doolittle index with a window size of 20 amino acids and a step size of 1 amino acid using GENETYX (version 10.1.1) (Genetyx, Tokyo, Japan).

### Elephant tissues and cell lines

Liver and muscle tissue samples were collected from an adult male of Asian elephant dead at Kobe Oji Zoo in Japan, and were stored at -80°C until use. Cell lines LACF-NANAI (RIKEN Cell Bank: RCB2319) and LACF-NANAII (RIKEN Cell Bank: RCB2320), which had been established from the gum and the ear of African elephant, respectively, were provided by RIKEN Cell Bank, Japan, and were maintained in Dulbecco’s modified Eagle’s medium (DMEM; GIBCO/BRL) containing 10% fetal bovine serum (FBS), L-glutamine, and penicillin-streptomycin under 5% CO_2_ at 37°C. The 293F cells (Invitrogen) were maintained in Eagle’s minimum essential medium (EMEM; GIBCO/BRL) containing 5% FBS, L-glutamine, and penicillin-streptomycin under 5% CO_2_ at 37°C. BHK-21 cells (RIKEN Cell Bank: RCB1423), which were provided by RIKEN Cell Bank, were maintained in DMEM containing 10% FBS, L-glutamine, and penicillin-streptomycin under 5% CO_2_ at 37°C.

### RT-PCR on elephant samples

Total RNA was extracted from the liver and muscle tissues of an Asian elephant; 50 mg of each tissue was frozen with beads (TOMY) and 1 ml of ISOGEN (Nippon Gene) in liquid nitrogen, and homogenized using TOMY Micro Smash MS-100R (TOMY). The homogenized sample was mixed with 200 μl of 100% chloroform, and the mixture was centrifuged at 12,000 × g for 15 min at 4°C. The supernatant with 600 μl of 70% ethanol was added to an RNeasy Spin Column provided in the RNeasy Mini Kit (QIAGEN), and RNA was extracted following manufacturer’s instructions. Total RNA was also extracted from LACF-NANAI, LACE-NANAII, Baby Hamster Kidney (BHK) cells, and 293F cells using an RNeasy Mini Kit according to manufacturer’s instructions. DNase digestion was performed on all samples during the extraction process using RNase-free DNase set (QIAGEN).

Expression of mRNAs containing laEBLN-1 and laEBLN-2 or their orthologues in the elephant samples was examined by RT-PCR. One-step RT-PCR was conducted using a SuperScript III/Platinum Taq one-step RT-PCR kit (Invitrogen) in a final volume of 25 μl containing 1 × reaction mixture, 0.4 μM primers, 1 μl SuperScript III RT/Platinum Taq Mix, and 20 ng of total RNA. Primer sequences were designed with Primer Blast in NCBI as listed in S5 Table. RT-PCR was performed as follows; reverse transcription at 50°C for 60 min, 40 cycles of 94°C for 30 sec, 60°C for 30 sec, and 72°C for 30–90 sec, followed by a final extension at 72°C for 3 min. In 2-step RT-PCR, cDNA was synthesized from mRNAs using oligodT primer with or without SuperScript III Reverse Transcriptase (Invitrogen), and the product was used as the template for PCR. In brief, PCR was performed in a final volume of 25 μl containing 1 × PCR Buffer, 0.2 mM dNTP, 0.4 μM primers, 1.25 U Blend Taq (TOYOBO), and 1μl of the above template. PCR reaction was performed as follows; 94°C for 2 min and 40 cycles of 94°C for 30 sec, 55°C for 30 sec, and 72°C for 30 sec. The size of the products was analyzed by agarose gel electrophoresis.

### 5’ and 3’ RACEs for laEBLN-1v1 and laEBLN-2v1

The 5’ and 3’ RACEs for laEBLN-1v1 and laEBLN-2v1 were performed using 2.75–3.75 μl of total RNA collected from LACF-NANAII with SMARTer RACE cDNA Amplification Kit (Clontech). Briefly, after incubation of the total RNA with 12 μM 5’ and 3’ CDS primers at 72°C for 3 min and at 42°C for 2 min, the first-strand cDNA synthesis for 3’ (5’) RACE was performed in a final volume of 10 μl containing 1 × First-Strand Buffer, 20 mM DTT, 10 mM dNTP mix, 10 U RNase Inhibitor, (12 μM SMARTer II A Oligonucleotide,) and 50 U SMARTScribe Reverse Transcriptase at 42°C for 90 min, followed by a termination process at 70°C for 10 min. The reaction mixture was diluted to 100 μl with Tricine-EDTA buffer, and was used as the template for PCR in a final volume of 25 μl containing 1 × PCR Buffer, 0.2 mM dNTP, 0.4 μM primers, 1.25 U Blend Taq (TOYOBO), and 1 μl of the above cDNA mixture. Nested-PCR was performed using the PCR products diluted 500-fold with distilled water, and the products were purified with Wizard SV Gel and PCR Clean-Up System (Promega). The nested-PCR products were ligated into the pGEM-T Easy vector (Promega) using T4 DNA ligase (New England Biolabs), and the plasmids were transformed into TOP10 Competent Cells (Life Technologies). For each of 5’ and 3’ RACEs, at least 10 colonies were selected by direct-colony PCR and the plasmids were purified from the colonies using the Wizard Plus SV Minipreps DNA Purification System (Promega). Nucleotide sequences of the inserts were determined using BigDye Terminator v3.1 Cycle Sequencing Kit with ABI Prism 3130 (ABI).

### Rabbit polyclonal antiserum against recombinant laEBLN-1v1 protein

Genomic DNA was extracted from LACF-NANAII using a QIAamp DNA Blood Mini Kit (QIAGEN) according to manufacturer’s instructions. The ORF of laEBLN-1v1 was amplified from genomic DNA with the primer pair AFEBLN_pET_F and AFEBLN_pET_R (S5 Table). The PCR product was ligated into the PET100 vector (Invitrogen) using Ligation high ver.2 (TOYOBO) at 16°C for 30 min, and the plasmid was transformed into BL21 cells. Colonies harboring the plasmids were selected and propagated in LB medium containing 0.02% lactose, 0.05% glucose, 0.5% glycerine, 2 mM MgSO_4_, and phosphate buffer for overexpression of the protein encoded by laEBLN-1v1. BL21 cells were disrupted using ultrasonic wave, and centrifuged at 6,000 × g for 30 min at 4°C. The recombinant laEBLN-1v1 (rlaEBLN-1v1)protein was purified from the supernatant with His-Trap HP and Hi-trap desalting column (GE Healthcare), and was used as the antigen for immune induction.

In the immune induction, the rlaEBLN-1v1 protein and Gold Titer Max (CytRx Corporation) were mixed in equal volumes, and 200 μl of the mixture containing 100 mg antigen was inoculated subcutaneously at four sites in the back of 90 day old rabbits (New Zealand White, female, SLC Japan). The intradermal injection was conducted four times within 1.5 months with intervals of 1 or 2 weeks. Whole blood was collected from the rabbits by cardiac puncture under anesthesia with pentobarbital Na (25 mg/kg), and the rabbits were euthanized with an overdose of pentobarbital Na (100 mg/kg). Increases in the antibody titers against rlaEBLN-1v1 protein in the rabbits were confirmed by ELISA. Rabbit polyclonal antiserum was collected from the whole blood and was used for western blot analysis and immunohistochemical staining.

### Ethics statement

The animal husbandry methods and experimental design were endorsed by the *Nihon University Animal Health Laboratory’s Animal Ethics Committee* (Permit number: AP14B087). This animal ethic has been established based on the “Law for Humane Treatment and Management of Animals” and “Standards Relating to the Care and Management of Laboratory”, administered by the Ministry of the Environment and “Fundamental Guidelines for Proper Conduct of Animal Experiment and Related Activities in Academic Research Institutions” administered by the Ministry of Education, Culture, Sports, Science and Technology in Japan.

### Construction of mammalian cell expression plasmids

cDNAs reverse-transcribed from laEBLN-1v1 and laEBLN-1v2 mRNAs were amplified by PCR using Blend taq (TOYOBO) with the primer pairs listed in S5 Table. The PCR product of laEBLN-1v1 was ligated into 3×FLAG-CMV-14 vector (SIGMA) using Ligation high ver.2 (TOYOBO) after digestion with the restriction enzymes *HindIII* and *BamHI* (New England Biolabs) at 37°C for 60 min. The PCR product of laEBLN-1v2 was ligated into V5-His-TOPO vector (Life Technologies) according to manufacture’s instructions. The plasmids transformed into TOP10 Competent Cells (Life Technologies) were collected using PureYield Plasmid Midiprep System (Promega) after confirming the insert with nucleotide sequencing. In addition, sub-cloning of ORFs in laEBLN-1v1 and laEBLN-1v2 into pcDNA3.10 vector (Thermo Fisher Scientific) was conducted using the above plasmids as templates for PCR, which were digested with *HindIII* and *BamHI* (New England Biolabs).

### Western blotting

The 293F cells were seeded onto 6 well plate at 5 × 10^5^ cells/well. On the following day, the above plasmids of 500 ng were transfected into the cells using ViaFect (Promega) according to manufacturer’s instructions, and after 24 hrs the whole cellular protein was extracted from the 293F cells. In addition, whole cellular proteins were extracted from LACF-NANAI, LACF-NANAII, and 293F cells propagated in 100 mm^2^ dishes. In brief, after washing cells propagated in 100 mm^2^ dishes or 6 well plates with physiological salt solution, RIPA buffer (Santa Cruz Biotechnology) was added to each dish and each well, and the cells were incubated on ice for 15 min. The cells were scraped into 1.5 ml tubes, and were again incubated on ice for 60 min. The cells were disrupted with ultrasonic wave, and centrifuged at 12,000 rpm for 10 min at 4°C. The concentration of total proteins collected from LACF-NANAI, LACF-NANAII, and 293F cells was quantified using the Pierce 660 nm Protein Assay Reagent (Thermo Scientific) with Quick Start Bovine Serum Albumin Standard (Bio-Rad) by Multiscan GO (Termo Scientific).

The whole celluer protein samples were boiled with SDS sample buffer, and electrophoresed in a 12% TGX Stain-Free Fastcast acrylamide Gel (Bio-Rad) at 20 mA for 60 min. After trans-blotting onto a PVDF membrane using Trans-Blot Turbo Transfer System (Bio-Rad), the membrane was blocked using BlockAce (DS Pharma Biomedical) for 60 min. The membrane was incubated with 1,000-fold diluted rabbit polyclonal antiserum and 2,000-fold diluted mouse anti-alpha-Tubulin monoclonal antibody (MBL) in Can Get Signal solution 1 (TOYOBO) overnight at 4°C. After washing in PBS with 0.2% Tween 20 (PBST), the membrane was incubated with 50,000-fold diluted peroxidase-conjugated anti-rabbit secondary antibody and 25,000-fold diluted peroxidase-conjugated anti-mouse secondary antibody (GE Healthcare) in Can Get Signal solution 2 (TOYOBO) for 60 min at room temperature. ECL signal on the membrane was detected with ECL select solution (GE Healthcare) using an ImageQuant LAS-4000 imaging system (GE Healthcare).

### Immunohistochemical staining

The LACF-NANAI and 293F cells were seeded onto a glass slide at 1 × 10^4^ and 2 × 10^5^ cells/slide (Matsunami Glass), and 500 ng of EBLN expression plasmids were transfected into the cells. After 48 hrs, these cells were fixed with 4% formaldehyde solution in PBS (PFA) for 15 min at room temperature. In addition, to observe subcellular localization of endogenous laEBLN proteins, LACF-NANAI and LACF-NANAII were harvested onto a glass slide, and fixed with 4% PFA for 15 min at room temperature. These cells were subjected to 0.1% triton in PBS for 10 min at room temperature. The 200-fold diluted rabbit polyclonal antiserum against laEBLN-1v1 protein and 1,000-fold diluted Alexa Fluor 594 Goat Anti-rabbit IgG antibody (Invitrogen) were used for detection of endogenous laEBLN proteins. In addition, 100-fold diluted Alexa Fluor 488 conjugated anti-KDEL polyclonal antibodies (Funakoshi) and 400-fold diluted Alexa Fluor 488 conjugated anti-S6 Ribosomal protein monoclonal antibody (Cell Signaling Technology) were used for detection of the ER and ribosomes in the cells, respectively. The 1,000-fold diluted Anti-DDDDK-tag mAb-Alexa Fluor 488 (MBL) and Anti-His-tag mAb-Alexa Fluor 488 (MBL) were used to detect FLAG-tagged laEBLN-1v1 and His-tagged laEBLN-1v2 proteins expressed in LACF-NANAI and 293F cells. Immunohistochemical imaging of the cells was performed using an Olympus Microscope IX71.

### Promoter assay

Genomic DNA was extracted from LACF-NANAII using a QIAamp DNA Blood Mini Kit (QIAGEN) according to manufacturer’s instructions. PCRs for cloning exon 1 and upstream regions of laEBLN-1v1/1v2 and laEBLN-2v1/2v2 was performed in a final volume of 25 μl containing 1 × PCR Buffer, 0.2 mM dNTP, 0.4 μM primers, 1.25 U Blend Taq (TOYOBO), and 1 μl of genomic DNA, and the PCR products were purified with a Wizard SV Gel and PCR Clean-Up System (Promega). After digestion of the PCR products and the firefly luciferase (pGL4.10) vector (Promega) with the restriction enzyme *KpnI*, *HindIII*, or *EcoRV* (New England Biolabs) at 37°C for 60 min, the PCR products were ligated into the vector using Ligation high ver.2 (TOYOBO) at 16°C for 30 min, and the plasmids were transformed into TOP10 Competent Cells (Life Technologies). Colonies containing plasmids were selected by direct-colony PCR, and plasmids were collected from the colonies using PureYield Plasmid Midiprep System (Promega).

The 293F cells were seeded onto 24 well plates at 5 × 10^3^ cells/well. On the following day, 100 ng of firefly luciferase plasmids containing exon 1 with or without upstream region of laEBLN-1v1/1v2 or laEBLN-2v1/2v2 was transfected into the cells using ViaFect (Promega) according to manufacturer’s instructions. As a normalization control, 10 ng of renilla luciferase (pRL-TK) vector (Promega) was also used for transfection. At 24 hrs after transfection, cells were lysed with Passive lysis buffer (Promega), and the luciferase activity was measured by a microplate luminometer (Centro LB 960, Berthold Technologies, Bad Wildbad, Germany) using a Dual-Luciferase Reporter Assay System (Promega). Activity values for firefly luciferase were normalized to those for renilla luciferase. The assay was conducted three times in tripricate wells.

## Supporting Information

S1 Fig Multiple alignment of amino acid sequences encoded by ORFs of cluster I EBLNs. Sequence names correspond to those in Fig 1A. Fragmented ORFs encoded by cluster I EBLNs of cape golden mole (Ca/AMDV01031468) were assigned to Ca/AMDV01031468a and Ca/AMDV01031468b.
(TIF)

S2 Fig Maximum likelihood tree of afrotherian EBLNs. Each sequence name corresponds to that in Fig 1A. Red-, blue-, and green-letters indicate the EBLNs belonging to cluster I, cluster II, and others, respectively, which were categorized according to the phylogenetic tree shown in Fig 1A. Bootstrap values > 70% are shown on interior branches.
(TIFF)

S3 Fig Dot-plots of nucleotide sequences between laEBLN-1v1 and other afrotherian EBLNs. Red boxes represent regions of afrotherian EBLNs homologous to bornavirus N identified by tBLASTn search. Green and red lines in the boxes indicate forward- and reverse-match positions between compared sequences, respectively. The nucleotide positions in the sequences used for the dot-plot analysis are listed in S7 Table.
(TIF)

S4 Fig Neighbor-joining tree of EBLNs identified in animal genomes. Each sequence is described as abbreviation of host species or virus/accession number (number of amino acid residues for cluster I EBLNs), except for laEBLN-1v1 and laEBLN-2v1. An: *Aotus nancymaae*, Cj: *Callithrix jacchus*, Cs: *Chlorocebus sabaeus*, Ccj: *Cebus capucinus imitator*, Cea: *Cercocebus atys*, Cap: *Colobus angolensis palliates*, Gg: *Gorilla gorilla*, Hs: *Homo sapiens*, Mf: *Macaca fascicularis*, Mm: *Macaca mulatta*, Mn: *Macaca nemestrina*, Ml: *Mandrillus leucophaeus*, NL: *Nasalis larvatus*, Nol: *Nomascus leucogenys*, Pp: *Pan paniscus*, Pt: *Pan troglodytes*, Paa: *Papio anubis*, Poa: *Pongo abelii*, Rr: *Rhinopithecus roxellana*, Sb: *Saimiri boliviensis*, Mim: *Microcebus murinus*, Dm: *Daubentonia madagascariensis*, Prc: *Propithecus coquereli*, Gv: *Galeopterus variegatus*, Min: *Miniopterus natalensis*, As: *Apodemus sylvaticus*, Cp: *Cavia porcellus*, Mum: *Mus musculus*, Ms: *Mus spretus*, Rn: *Rattus norvegicus*, Cc: *Condylura cristata*, Pm: *Python molurus*, Cmp: *Crotalus mitchellii Pyrrhus*, It: *Ictidomys tridecemlineatus*, La: *Loxodonta Africana*, Tm: *Trichechus manatus latirostris*, Pc: *Procavia capensis*, Ca: *Chrysochloris asiatica*, Oa: *Orycteropus afer*, and Ee: *Elephantulus edwardii*. Green-, orange-, brown-, purple, red-, cyan- and pink-letters are used for describing the EBLNs in primate (haplorrhini), primate (strepsirrhini), bat, snake, afrotheria, mole, and rodent, respectively. Bootstrap values > 70% are shown for interior branches.
(TIF)

S5 Fig Pairwise alignment of amino acid sequences between bornavirus N and ORF encoded by laEBLN-1v1. Functional domains characterized in bornavirus N are surrounded by dotted-lines. NLS: nuclear localization signal, PBS: P-binding site, NES: nuclear export signal [32]. The arrows indicate the sequence region where bornavirus N domain was predicted by Pfam domain search.
(TIFF)

S6 Fig Gene structures at EBLN loci in the genome of Florida manatee. Red, blue and green arrows represent genomic locations of EBLNs, TMEM106B and SCIN in the genome of *Trichechus manatus latirostris* (TriManLat1.0, JH594848), respectively. Numbers under the arrows indicate the nucleotide positions homologous to elephant TMEM106B and SCIN, and Tm/AHIN01338425 and Tm/AHIN01338426.
(TIFF)

S7 Fig Syntenic relationships between the genomes of African elephant and human. Homologous regions between African elephant and human were identified by tBLASTn (A) and BLASTn search (B). Each arrow represents the gene locus annotated in the genomes of *L*. *africana* (Loxafr3.0, scaffold 5) and *H*. *sapiens* (GRch38, Chr7).
(TIF)

S8 Fig Map of primers used for RT-PCR to amplify mRNAs transcribed from laEBLN-1 and laEBLN-2 loci. The arrows represent the target positions of primers in laEBLN mRNAs. Nucleotide sequences of primers a-h are shown in S5 Table. The primer pairs used for RT-PCR are connected with dotted-lines: Primer pairs a/b, c/e, d/e, c/f, and d/f were used to amplify all laEBLNs, laEBLN-1v1, laEBLN-1v2, laEBLN-2v1, and laEBLN-2v2, respectively, by one-step RT-PCR. The primer pair g/h was used for two-step RT-PCR after cDNA synthesis with oligodT primer.
(TIF)

S9 Fig Expression of laEBLNs mRNA in elephant tissues and cell lines. PCR was conducted using cDNA products synthesized from RNA extracts of LACF-NANAI, LACF-NANAII, Asian elephant liver and muscle tissues after reverse transcription with or without reverse transcriptase (RT).
(TIF)

S10 Fig Dot-plot between nucleotide sequences of laEBLN-1v1 and laEBLN-2v1 mRNAs with 1,500 nt upstream region. Each arrow represents the exons of laEBLNs. Coding and non-coding regions in exons are shown in red and pink, respectively. Green and red lines in the box indicate forward and reverse match positions between compared sequences, respectively.
(TIF)

S11 Fig Multiple alignment of amino acid sequences of laEBLNs. Amino acid sequences surrounded by a red-box indicate those encoded by exon 3 of laEBLN-1v2 and laEBLN-2v2 mRNAs.
(TIF)

S12 Fig Cellular localization of laEBLN-1v1 and laEBLN-1v2 proteins expressed in 293F cell. Immunohistochemical staining of (A) laEBLN-1v1 and (B) laEBLN-1v2 expressed in 293F cell. First-antibodies or DAPI used for immunohistochemical staining are shown in parenthesis.
(TIF)

S13 Fig Multiple alignment of homologous sequences among laEBLN-1, laEBLN-2, and TMEM106B. Multiple alignment of nucleotide sequences in exon 1 and the upstream regions of laEBLN-1v1, laEBLN-2v1, and TMEM106B. Red arrows indicate transcription start sites of the above mRNAs.
(TIF)

S14 Fig Multiple alignment around 5’ splice donor site among laEBLN-1, laEBLN-2, and TMEM106B. Red and black letters indicate the nucleotide sequences in exon 1 and intron, respectively. 5’ splice donor sites used for the splicing in TMEM106B, laEBLN-1 and laEBLN-2 are surrpunded by a red-line.
(TIFF)

S15 Fig Mapping information of TMEM106B mRNA in the human genome (GRch38, Chr7). These data were obtained from DBTSS [27] on December 23, 2015.
(TIFF)

S1 Table EBLNs identified in afrotherian genomes by tBLASTn search using bornavirus N as a query. ^a^Accession numbers in bold fonts show the cluster I EBLNs categorized from the phylogenetic tree in Fig 1A. ^b^Amino acid positions of bornavirus N (accession number: AY374520). ^c^EBLN sequence identified in ALYB01141343 was not used for the phylogenetic analyses because of no overlap region with other EBLNs.
(XLSX)

S2 Table *d*_N_/*d*_S_ ratios estimated for the ORFs encoded by cluster I EBLNs. ^a^a-q correspond to the branches of the phylogenetic tree shown in Fig 1C. ^b^p-values were calculated by the likelihood ratio test.
(XLSX)

S3 Table Nucleotide positions of laEBLNs in *L*. *africana* genome. ^a^Number in parentheses indicate the nucleotide positions of CDS encoded by laEBLNs.
(XLSX)

S4 Table Genomic loci of TMEM106B and SCIN in animal genomes. ^a^Genome locations orthologous to transcript variant 1 of TMEM106B and SCIN were refered from the NCBI databse on 4^th^ December 2015.
(XLSX)

S5 Table Sequence information of primers used for the present study. ^a^Primer map used for RT-PCR in Fig 3 and S9 Fig are shown in S8 Fig.
(XLSX)

S6 Table Celluer localization of laEBLNs predicted by PSPORT II [22]. (XLSX)

S7 Table Nucleotide positions of the sequences used for dot-plot analysis in S3 Fig. (XLSX)

S1 File Multiple alignment of amino acid sequences of EBLNs and bornavirus N used for the phylogenetic analyses. Each equence name corresponds to that in Fig 1A, S2 and S4 Figs.
(FASTA)
