# Supplementary figures and images for: Exaptation of Bornavirus-Like Nucleoprotein Elements in Afrotherians
Source: PLoS Pathog. 2016 Aug 12;12(8):e1005785. doi: 10.1371/journal.ppat.1005785 (PMC4982594; doi:10.1371/journal.ppat.1005785)

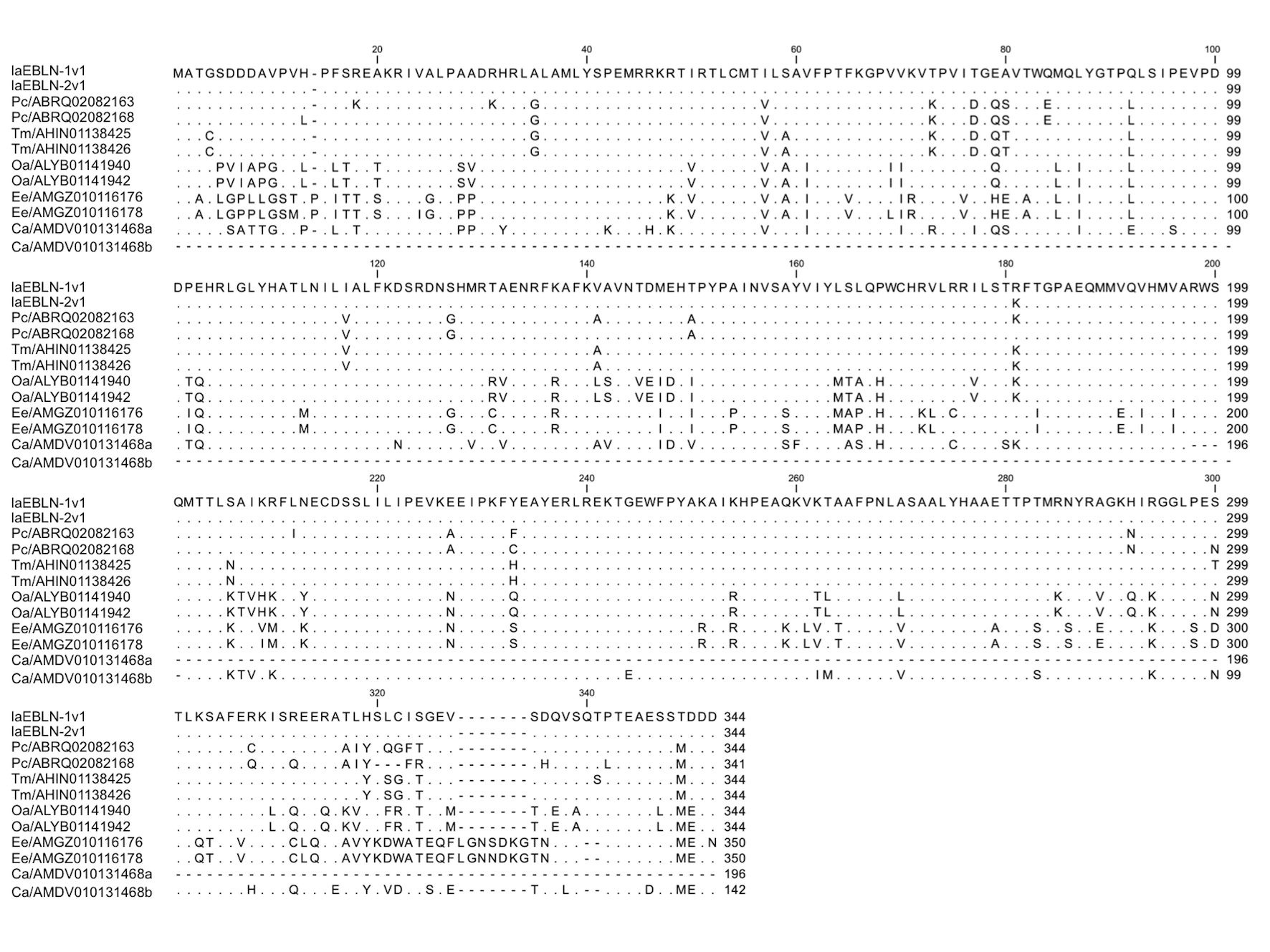

Supplement: S1 Fig — Sequence names correspond to those in Fig 1A. Fragmented ORFs encoded by cluster I EBLNs of cape golden mole (Ca/AMDV01031468) were assigned to Ca/AMDV01031468a and Ca/AMDV01031468b. (TIF) [file ppat.1005785.s001.tif]

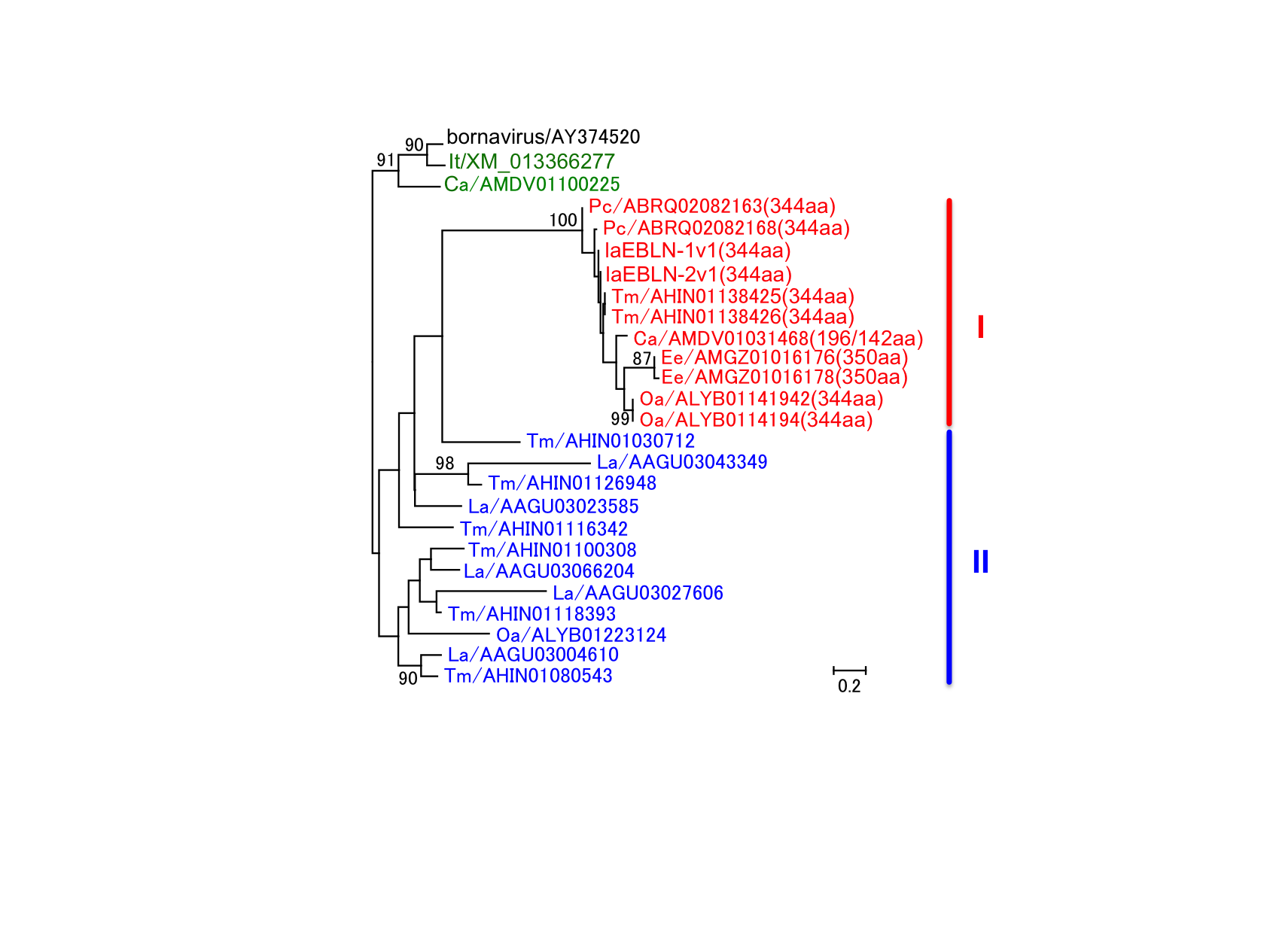

Supplement: S2 Fig — Each sequence name corresponds to that in Fig 1A. Red-, blue-, and green-letters indicate the EBLNs belonging to cluster I, cluster II, and others, respectively, which were categorized according to the phylogenetic tree shown in Fig 1A. Bootstrap values > 70% are shown on interior branches. (TIFF) [file ppat.1005785.s002.tiff]

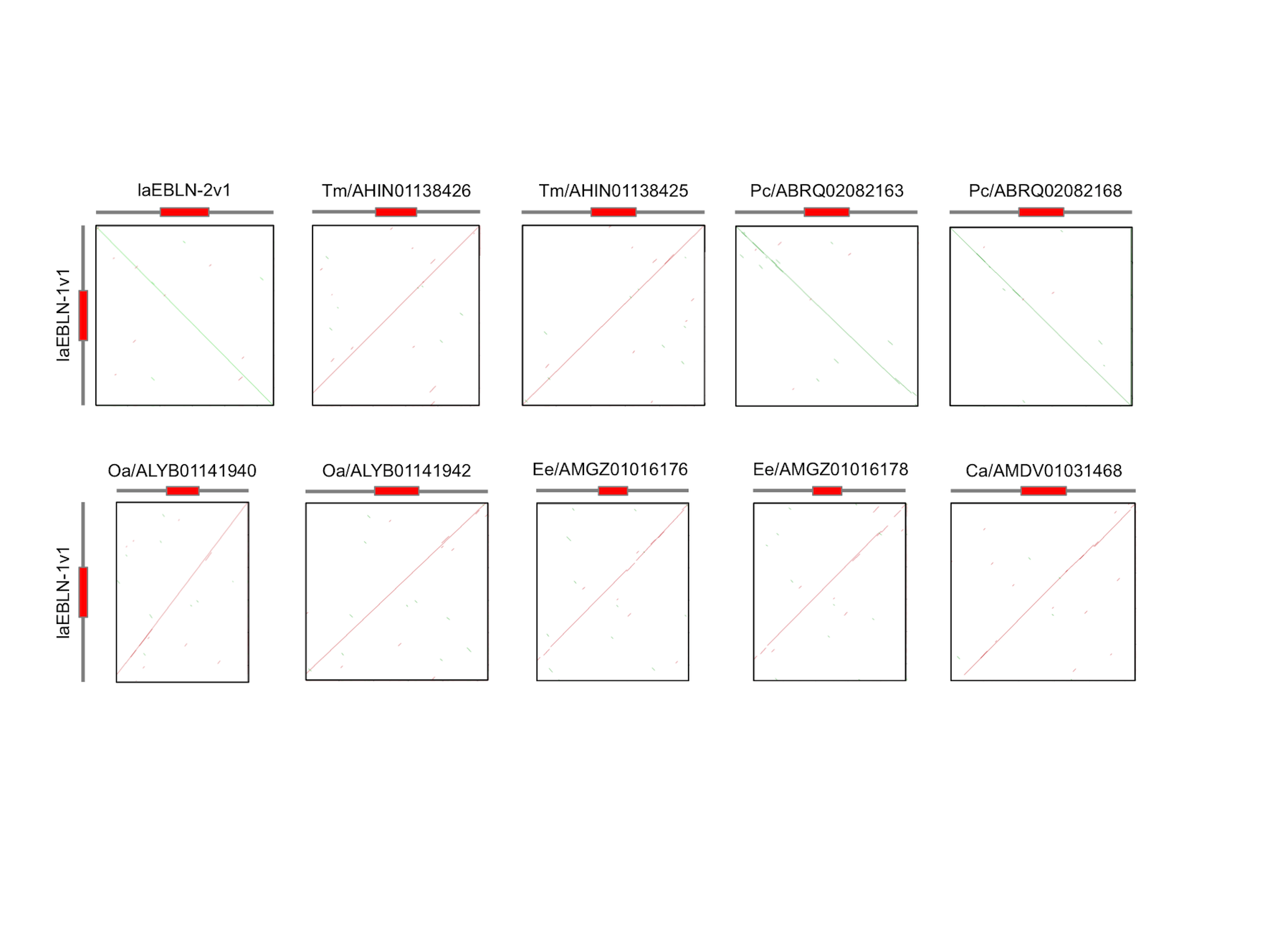

Supplement: S3 Fig — Red boxes represent regions of afrotherian EBLNs homologous to bornavirus N identified by tBLASTn search. Green and red lines in the boxes indicate forward- and reverse-match positions between compared sequences, respectively. The nucleotide positions in the sequences used for the dot-plot analysis are listed in S7 Table. (TIF) [file ppat.1005785.s003.tif]

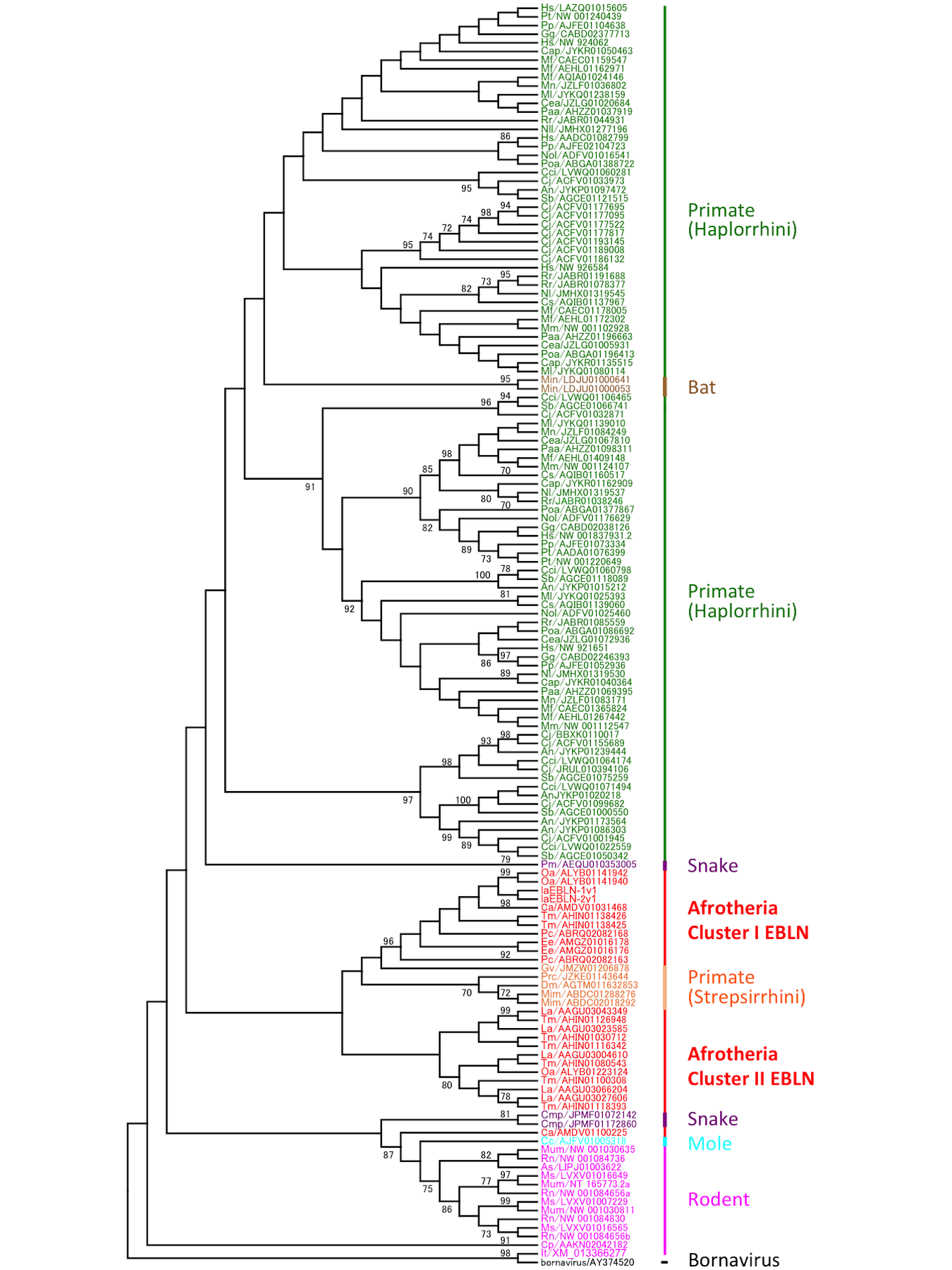

Supplement: S4 Fig — Each sequence is described as abbreviation of host species or virus/accession number (number of amino acid residues for cluster I EBLNs), except for laEBLN-1v1 and laEBLN-2v1. An: Aotus nancymaae, Cj: Callithrix jacchus, Cs: Chlorocebus sabaeus, Ccj: Cebus capucinus imitator, Cea: Cercocebus atys, Cap: Colobus angolensis palliates, Gg: Gorilla gorilla, Hs: Homo sapiens, Mf: Macaca fascicularis, Mm: Macaca mulatta, Mn: Macaca nemestrina, Ml: Mandrillus leucophaeus, NL: Nasalis larvatus, Nol: Nomascus leucogenys, Pp: Pan paniscus, Pt: Pan troglodytes, Paa: Papio anubis, Poa: Pongo abelii, Rr: Rhinopithecus roxellana, Sb: Saimiri boliviensis, Mim: Microcebus murinus, Dm: Daubentonia madagascariensis, Prc: Propithecus coquereli, Gv: Galeopterus variegatus, Min: Miniopterus natalensis, As: Apodemus sylvaticus, Cp: Cavia porcellus, Mum: Mus musculus, Ms: Mus spretus, Rn: Rattus norvegicus, Cc: Condylura cristata, Pm: Python molurus, Cmp: Crotalus mitchellii Pyrrhus, It: Ictidomys tridecemlineatus, La: Loxodonta Africana, Tm: Trichechus manatus latirostris, Pc: Procavia capensis, Ca: Chrysochloris asiatica, Oa: Orycteropus afer, and Ee: Elephantulus edwardii. Green-, orange-, brown-, purple, red-, cyan- and pink-letters are used for describing the EBLNs in primate (haplorrhini), primate (strepsirrhini), bat, snake, afrotheria, mole, and rodent, respectively. Bootstrap values > 70% are shown for interior branches. (TIF) [file ppat.1005785.s004.tif]

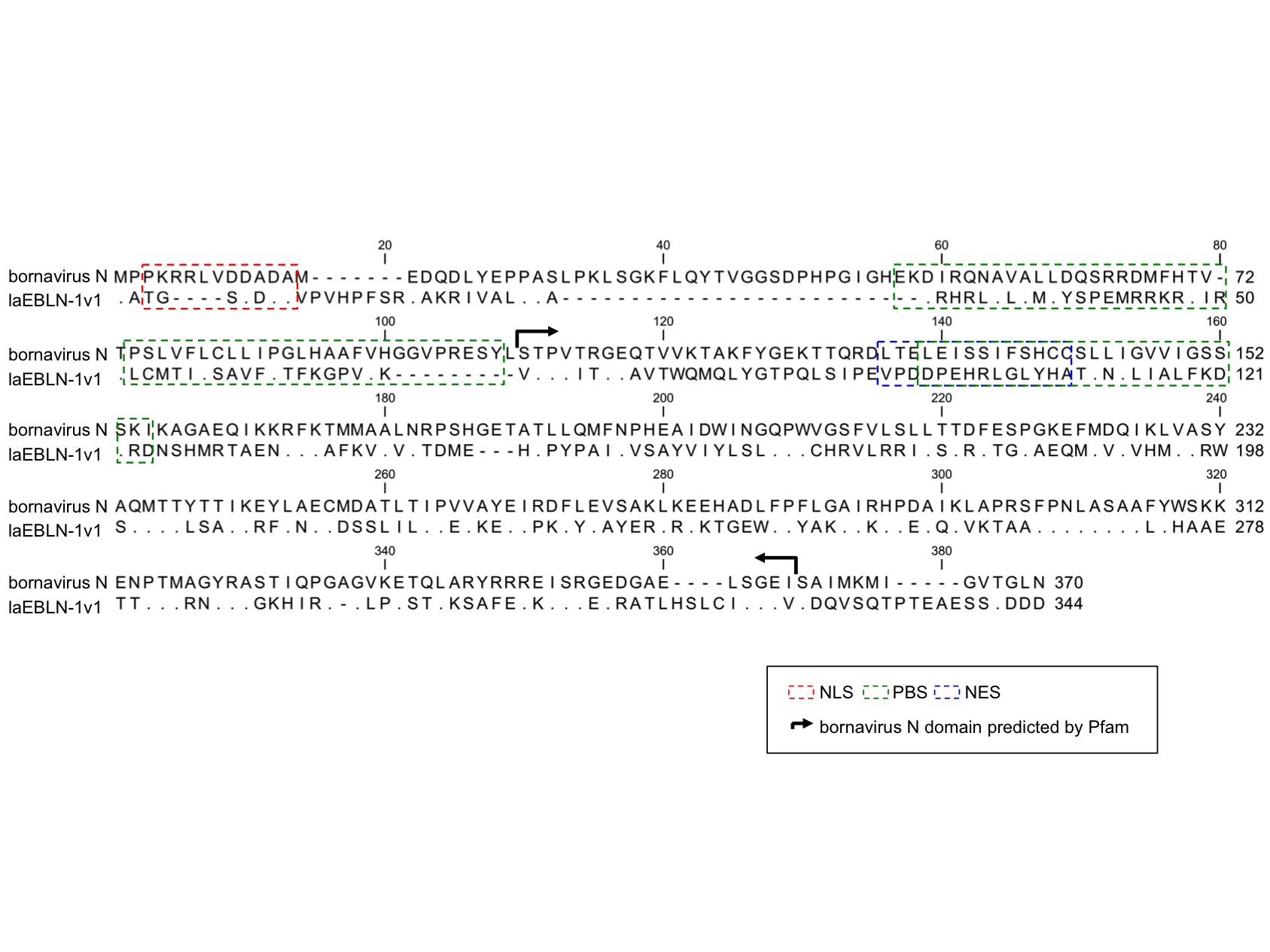

Supplement: S5 Fig — Functional domains characterized in bornavirus N are surrounded by dotted-lines. NLS: nuclear localization signal, PBS: P-binding site, NES: nuclear export signal [32]. The arrows indicate the sequence region where bornavirus N domain was predicted by Pfam domain search. (TIFF) [file ppat.1005785.s005.tiff]

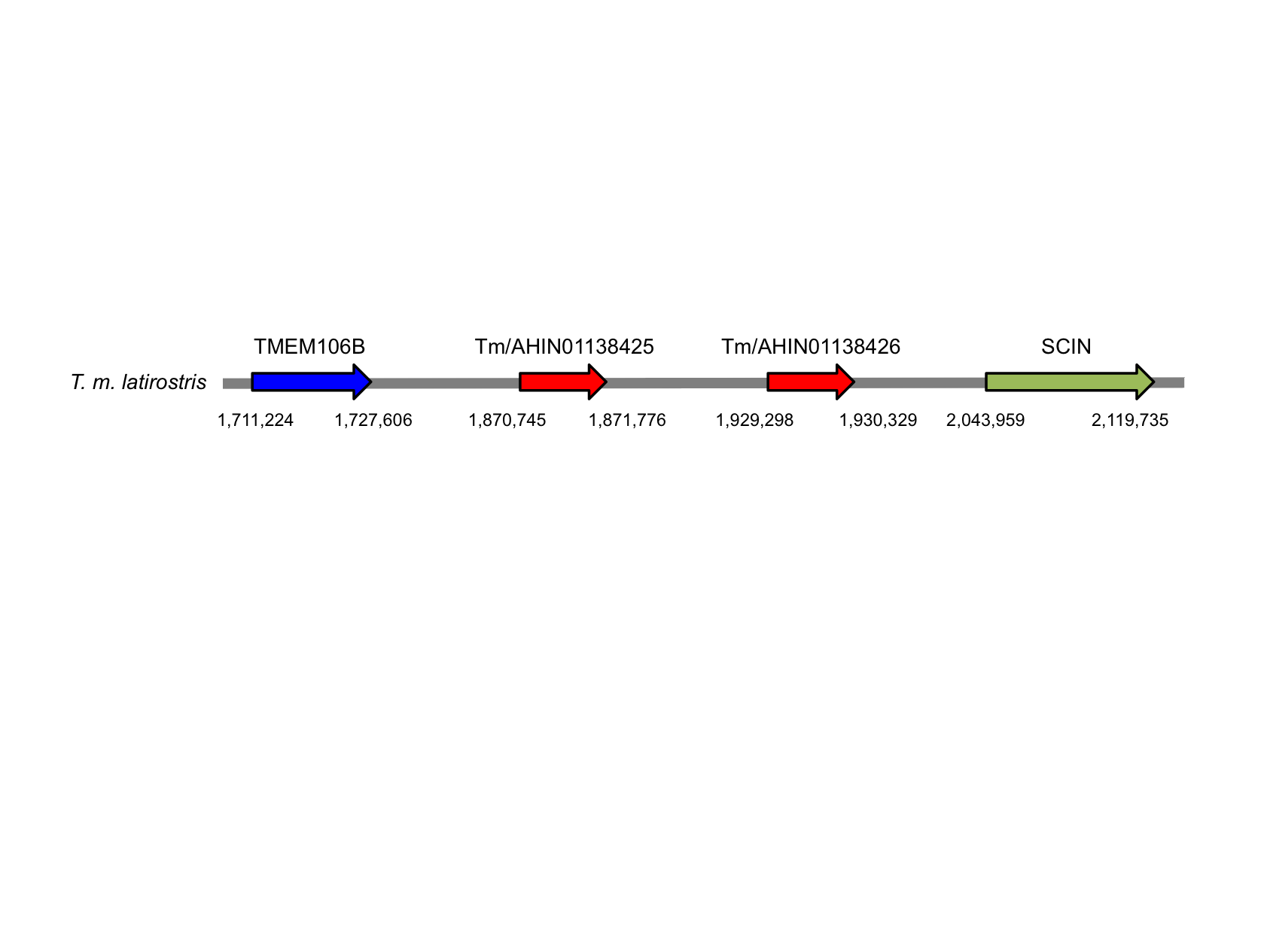

Supplement: S6 Fig — Red, blue and green arrows represent genomic locations of EBLNs, TMEM106B and SCIN in the genome of Trichechus manatus latirostris (TriManLat1.0, JH594848), respectively. Numbers under the arrows indicate the nucleotide positions homologous to elephant TMEM106B and SCIN, and Tm/AHIN01338425 and Tm/AHIN01338426. (TIFF) [file ppat.1005785.s006.tiff]

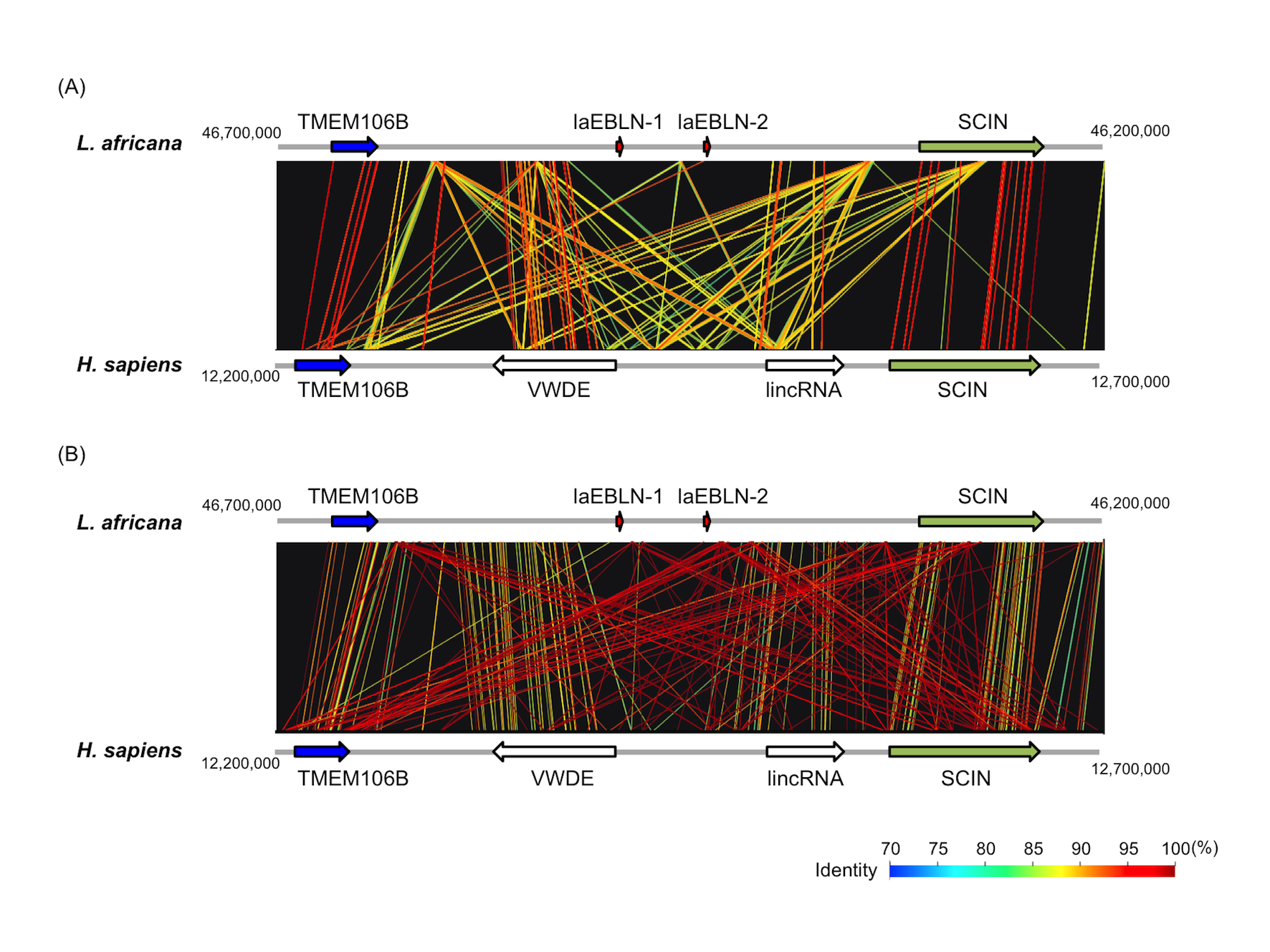

Supplement: S7 Fig — Homologous regions between African elephant and human were identified by tBLASTn (A) and BLASTn search (B). Each arrow represents the gene locus annotated in the genomes of L. africana (Loxafr3.0, scaffold 5) and H. sapiens (GRch38, Chr7). (TIF) [file ppat.1005785.s007.tif]

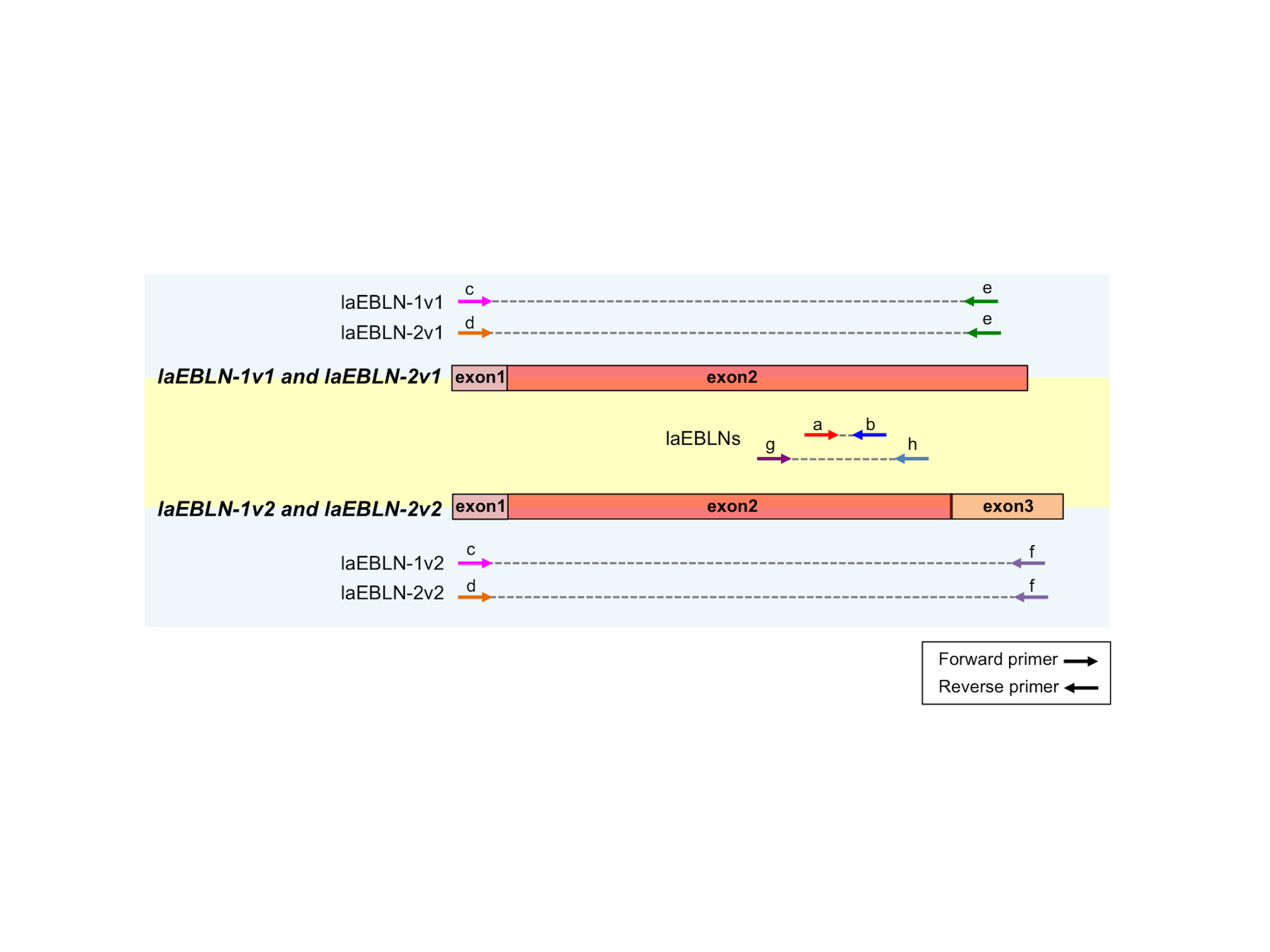

Supplement: S8 Fig — The arrows represent the target positions of primers in laEBLN mRNAs. Nucleotide sequences of primers a-h are shown in S5 Table. The primer pairs used for RT-PCR are connected with dotted-lines: Primer pairs a/b, c/e, d/e, c/f, and d/f were used to amplify all laEBLNs, laEBLN-1v1, laEBLN-1v2, laEBLN-2v1, and laEBLN-2v2, respectively, by one-step RT-PCR. The primer pair g/h was used for two-step RT-PCR after cDNA synthesis with oligodT primer. (TIF) [file ppat.1005785.s008.tif]

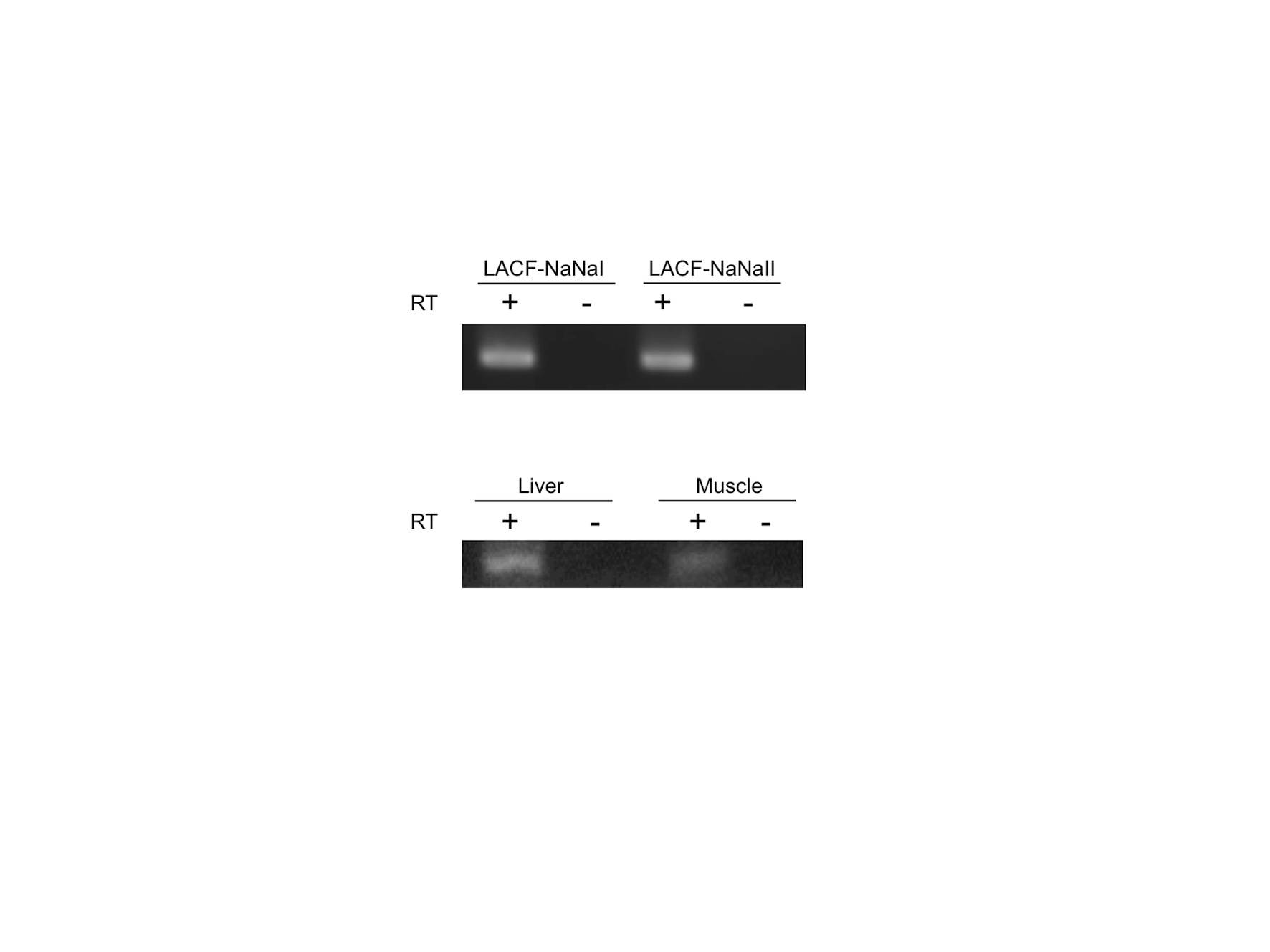

Supplement: S9 Fig — PCR was conducted using cDNA products synthesized from RNA extracts of LACF-NANAI, LACF-NANAII, Asian elephant liver and muscle tissues after reverse transcription with or without reverse transcriptase (RT). (TIF) [file ppat.1005785.s009.tif]

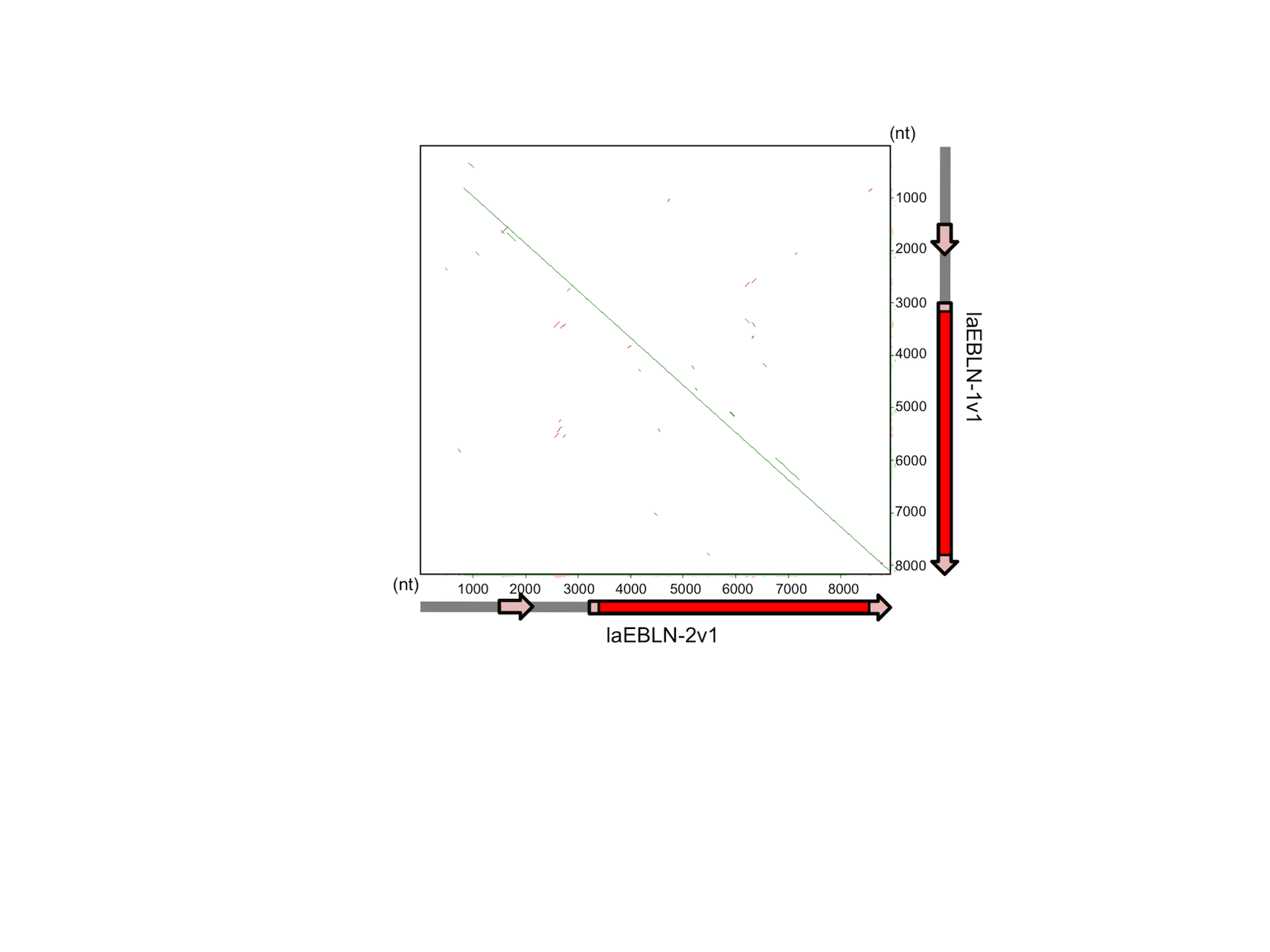

Supplement: S10 Fig — Each arrow represents the exons of laEBLNs. Coding and non-coding regions in exons are shown in red and pink, respectively. Green and red lines in the box indicate forward and reverse match positions between compared sequences, respectively. (TIF) [file ppat.1005785.s010.tif]

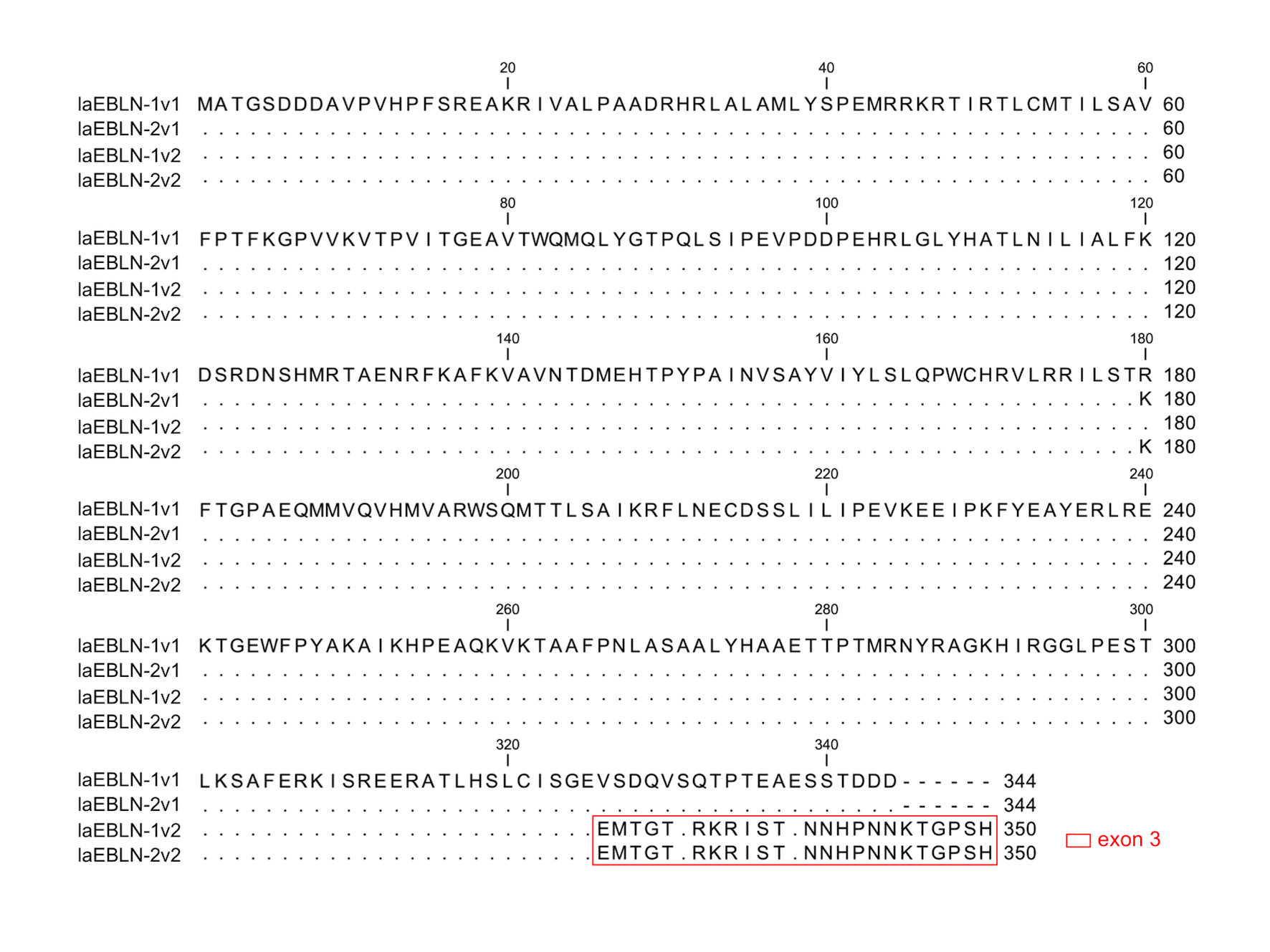

Supplement: S11 Fig — Amino acid sequences surrounded by a red-box indicate those encoded by exon 3 of laEBLN-1v2 and laEBLN-2v2 mRNAs. (TIF) [file ppat.1005785.s011.tif]

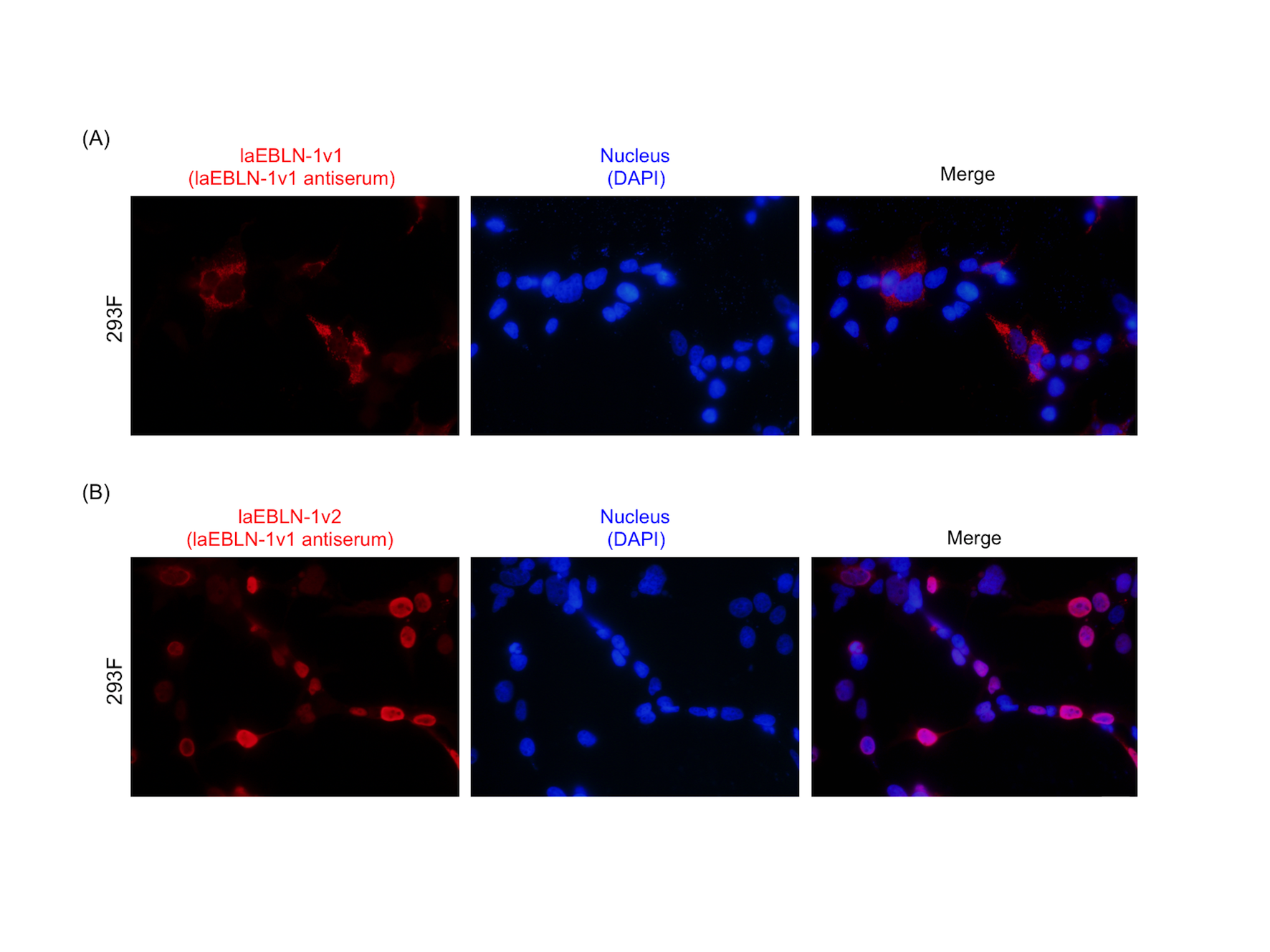

Supplement: S12 Fig — Immunohistochemical staining of (A) laEBLN-1v1 and (B) laEBLN-1v2 expressed in 293F cell. First-antibodies or DAPI used for immunohistochemical staining are shown in parenthesis. (TIF) [file ppat.1005785.s012.tif]

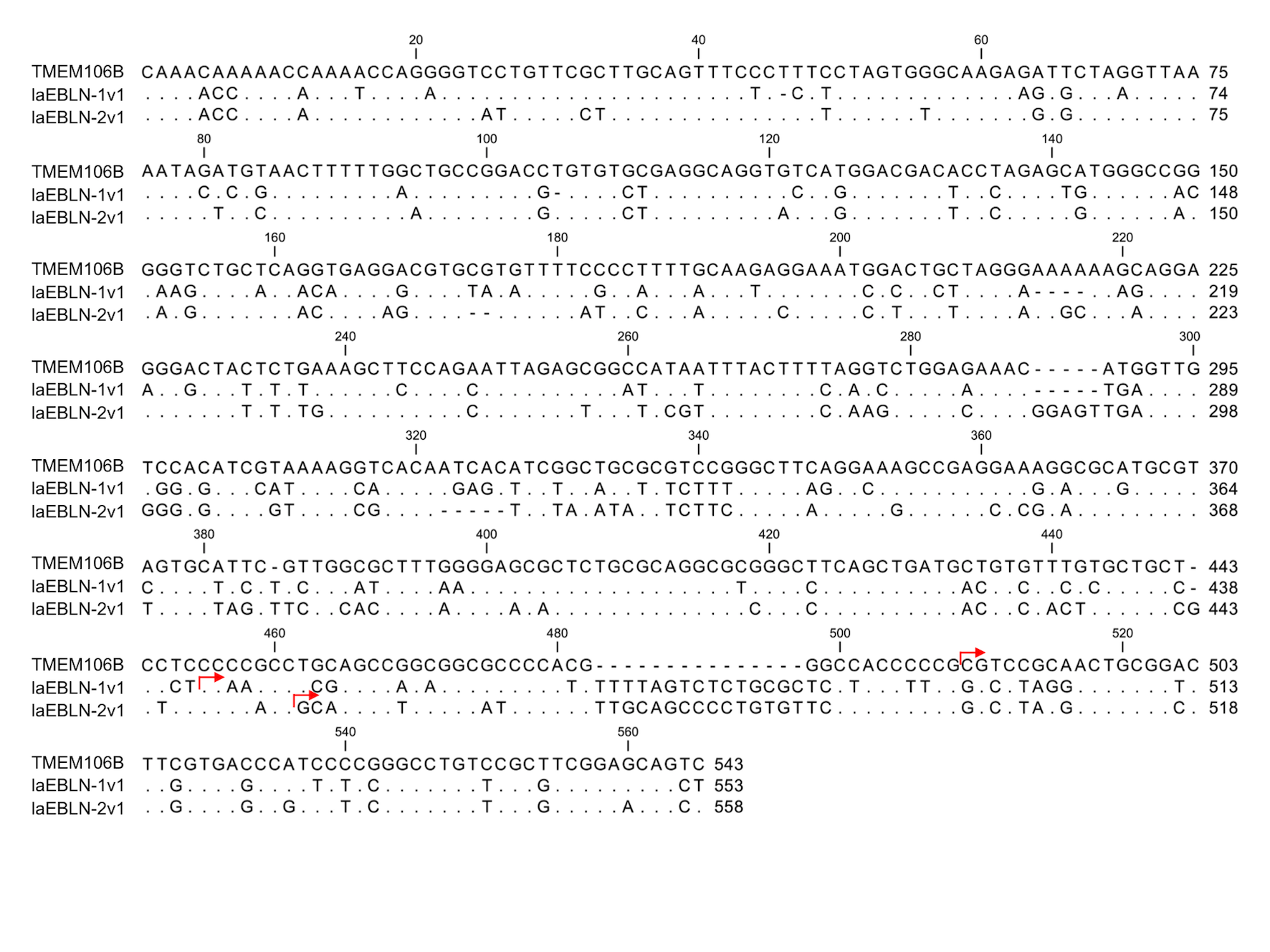

Supplement: S13 Fig — Multiple alignment of nucleotide sequences in exon 1 and the upstream regions of laEBLN-1v1, laEBLN-2v1, and TMEM106B. Red arrows indicate transcription start sites of the above mRNAs. (TIF) [file ppat.1005785.s013.tif]

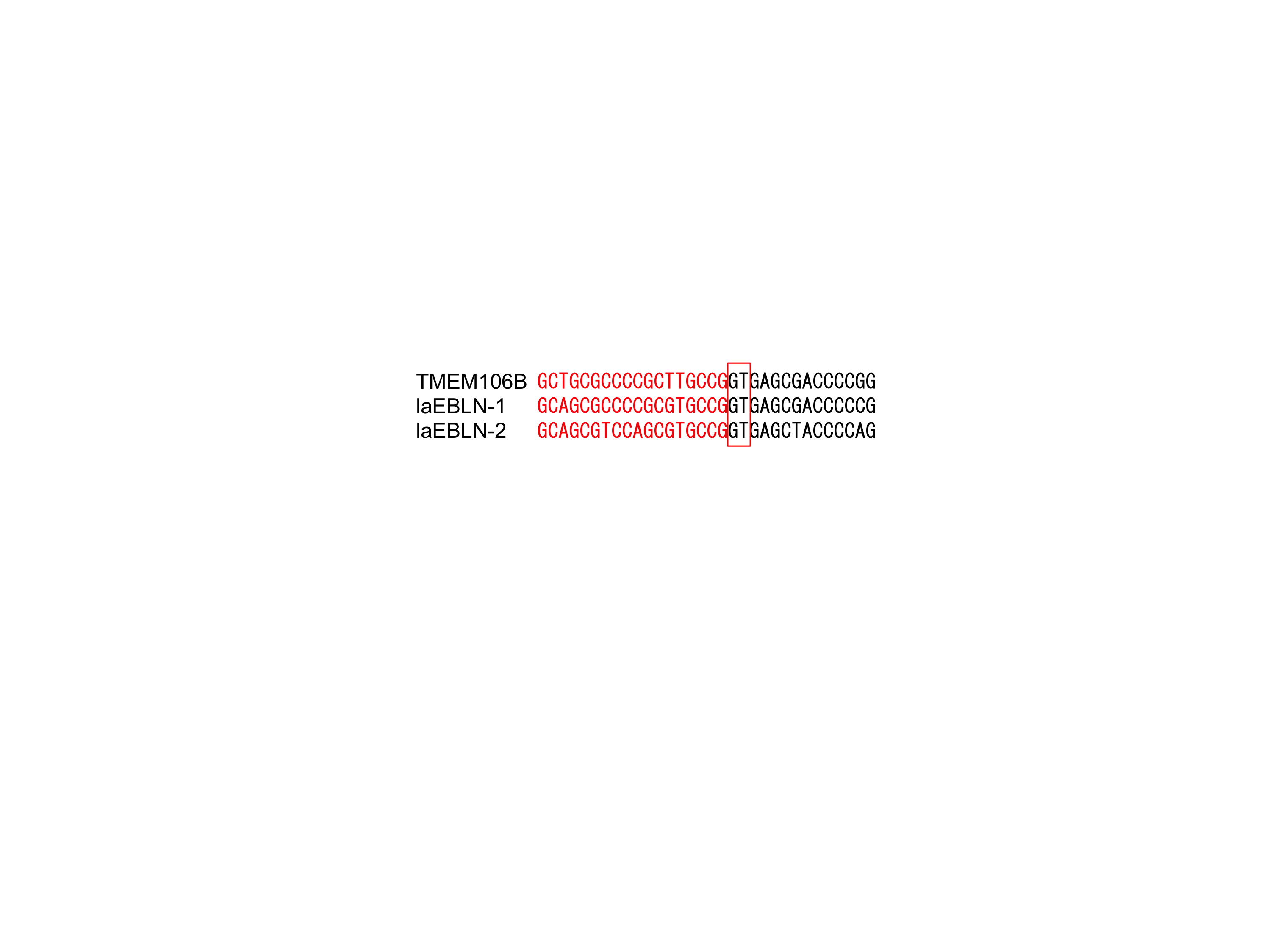

Supplement: S14 Fig — Red and black letters indicate the nucleotide sequences in exon 1 and intron, respectively. 5’ splice donor sites used for the splicing in TMEM106B, laEBLN-1 and laEBLN-2 are surrpunded by a red-line. (TIFF) [file ppat.1005785.s014.tiff]

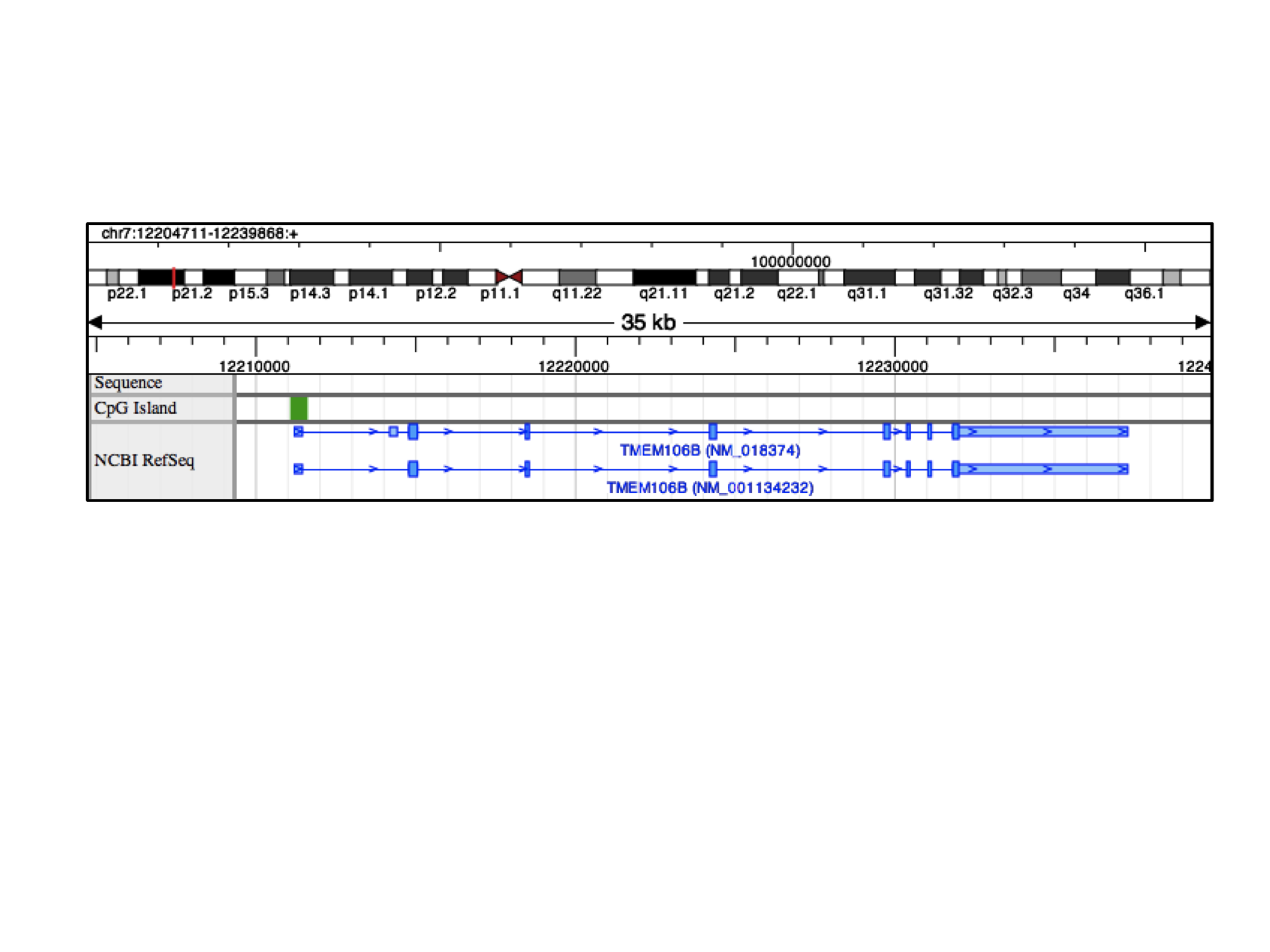

Supplement: S15 Fig — These data were obtained from DBTSS [27] on December 23, 2015. (TIFF) [file ppat.1005785.s015.tiff]
